# NKRF in Cardiac Fibroblasts Protects against Cardiac Remodeling Post‐Myocardial Infarction via Human Antigen R

**DOI:** 10.1002/advs.202303283

**Published:** 2023-09-05

**Authors:** Chenghu Guo, Wei Ji, Wei Yang, Qiming Deng, Tengfei Zheng, Zunzhe Wang, Wenhai Sui, Chungang Zhai, Fangpu Yu, Bo Xi, Xiao Yu, Feng Xu, Qunye Zhang, Wencheng Zhang, Jing Kong, Meng Zhang, Cheng Zhang

**Affiliations:** ^1^ National Key Laboratory for Innovation and Transformation of Luobing Theory The Key Laboratory of Cardiovascular Remodeling and Function Research Chinese Ministry of Education Chinese National Health Commission and Chinese Academy of Medical Sciences Department of Cardiology Qilu Hospital of Shandong University Jinan 250012 China; ^2^ Department of Ultrasonography Affiliated Hospital of Shandong University of Traditional Chinese Medicine Jinan 250014 China; ^3^ Department of Geriatric Cardiology Shandong Provincial Hospital Affiliated to Shandong First Medical University Jinan 250021 China; ^4^ Department of Epidemiology School of Public Health Cheeloo College of Medicine Shandong University Jinan 250012 China; ^5^ Key Laboratory Experimental Teratology of the Ministry of Education Department of Physiology School of Basic Medical Sciences Cheeloo College of Medicine Shandong University Jinan 250012 China; ^6^ Department of Emergency Medicine Chest Pain Center Shandong Provincial Clinical Research Center for Emergency and Critical Care Medicine Qilu Hospital Shandong University Jinan 250012 China; ^7^ Cardiovascular Disease Research Center of Shandong First Medical University Central Hospital Affiliated to Shandong First Medical University Jinan 250013 China

**Keywords:** cardiac fibroblasts, human antigen R, myocardial infarction, NF‐κB‐repressing factor, transcriptional and post‐transcriptional regulation

## Abstract

Myocardial infarction (MI) remains the leading cause of death worldwide. Cardiac fibroblasts (CFs) are abundant in the heart and are responsible for cardiac repair post‐MI. NF‐κB‐repressing factor (NKRF) plays a significant role in the transcriptional inhibition of various specific genes. However, the NKRF action mechanism in CFs remains unclear in cardiac repair post‐MI. This study investigates the NKRF mechanism in cardiac remodeling and dysfunction post‐MI by establishing a CF‐specific NKRF‐knockout (NKRF‐CKO) mouse model. NKRF expression is downregulated in CFs in response to pathological cardiac remodeling in vivo and TNF‐α in vitro. NKRF‐CKO mice demonstrate worse cardiac function and survival and increased infarct size, heart weight, and MMP2 and MMP9 expression post‐MI compared with littermates. NKRF inhibits CF migration and invasion in vitro by downregulating MMP2 and MMP9 expression. Mechanistically, NKRF inhibits human antigen R (HuR) transcription by binding to the classical negative regulatory element within the *HuR* promoter via an NF‐κB‐dependent mechanism. This decreases HuR‐targeted *M* *mp* *2* and *M* *mp* *9* mRNA stability. This study suggests that NKRF is a therapeutic target for pathological cardiac remodeling.

## Graphical abstract

Myocardial infarction (MI) is a leading global cause of mortality. Cardiac fibroblasts (CFs) are crucial for post‐MI cardiac repair. This study investigates the role of NF‐κB‐repressing factor (NKRF) in CFs during post‐MI remodeling, revealing its regulation of MMP2 and MMP9 expression and influence on CF migration and invasion. NKRF emerges as a potential therapeutic target to improve cardiac function post‐MI.

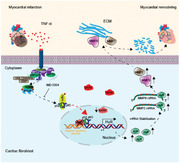

## 1 Introduction

Cardiac remodeling is the common pathological outcome of almost all cardiovascular diseases that lead to cardiac dysfunction and increased morbidity of heart failure and mortality.^[^ ^1^ ^]^ Ischemic heart disease and heart failure caused by myocardial infarction (MI) remain the leading causes of death worldwide.^[^ ^2^ ^]^ Scar formation post‐MI plays an important role in preventing ventricular rupture at an early stage and pumping function at a late stage. The extracellular matrix (ECM) serves as a mechanical scaffold to transmit signals under physiological conditions and participates in scar formation, cardiac remodeling, and cardiac function maintenance under pathological conditions.^[^ ^3^ ^]^ ECM homeostasis is maintained by changes in the balance between the secretion of collagen and activation of matrix metalloproteinases (MMPs) and tissue inhibitors of MMPs (TIMPs).^[^ ^1^, ^3^ ^]^ Cardiac fibroblasts (CFs) are the most abundant of all heart cell types and play an important role in regulating ECM.^[^ ^1^, ^4^ ^]^

Cardiac repair post‐MI is a finely orchestrated and complex series of events that can be roughly divided into the early inflammation/necrosis stage and the late fibrosis/proliferation stage.^[^ ^3^, ^5^ ^]^ A multitude of inflammatory cells (including neutrophils and macrophages) release many inflammatory factors in the early inflammation/necrosis phase and activate and secrete MMPs to digest and clear damaged cells and ECM tissues.^[^ ^3c^ ^]^ The released MMPs affect the homeostasis of ECM by disrupting the collagen fibers and struts in the ECM.^[^ ^6^ ^]^ Many CFs migrate to the infarct region, differentiate into myofibroblasts, and secrete a large amount of procollagen to promote scar formation and infarct repair in the late fibrosis/proliferation phase.^[^ ^3^, ^4^, ^7^ ^]^ Appropriate and timely restriction of the inflammatory degree and duration in the early inflammation/necrosis phase are determinants of the quality of wound healing in the late fibrosis/proliferation phase.^[^ ^8^ ^]^ Most previous studies focused solely on macrophages and cardiomyocytes (CMs) in the early inflammation/necrosis phase or the effect of CFs on cardiac remodeling after MI in the late fibrosis/proliferation phase.^[^ ^9^ ^]^ More research is needed to understand how inflammation affects cardiac function post‐MI by exploring the molecular determinants of CF‐derived ECM remodeling.

NF‐κB‐repressing factor (NKRF) is a transcription‐repressing factor encoded by a gene on the X chromosome and is expressed in many tissues including the heart, brain, lung, liver, kidney, and intestine.^[^ ^10^ ^]^ Endogenous NKRF is mainly directly bound to NF‐κB, which inhibits the expression of partial NF‐κB target genes, such as interferon‐β (*IFN‐β*), interleukin‐8 (*IL‐8*), and inducible nitric oxide synthase (*iNOs*).^[^ ^11^ ^]^ Increased oxidative stress in peripheral blood mononuclear cells (PBMCs) from patients with chronic obstructive pulmonary disease inhibit NKRF expression and impair its negative regulatory mechanism, which further aggravates IL‐8 production.^[^ ^12^ ^]^ Upregulation of NKRF expression in PBMCs and alveolar macrophages is observed in patients with pulmonary tuberculosis. NKRF binds to the negative regulatory element (NRE) of *IP‐10* and *IL‐8* promoters and inhibits the binding of NF‐κB and RNA polymerase II. This results in the downregulation of IP‐10 and IL‐8 expression.^[^ ^13^ ^]^ NKRF also inhibits the expression of monocyte chemoattractant protein‐1 (MCP‐1) in visceral adipose cells.^[^ ^14^ ^]^ These results suggest that NKRF plays a role in the transcriptional inhibition of various specific genes via the NRE. Recent studies have highlighted the multifaceted role of NKRF in various pathological conditions. A highly expressed long noncoding RNA Uc003xsl.1 directly binds NKRF in triple‐negative breast cancer; this disrupts its negative regulation of the NF‐κB‐responsive gene IL‐8 and promotes tumor progression and metastasis.^[^ ^15^ ^]^ Additionally, miRNA‑301a‑3p enhances tumor invasion and migration by targeting NKRF in human gastric cancer, leading to the activation of NF‐κB signaling and influencing patient prognosis.^[^ ^16^ ^]^ These findings underscore the significance of NKRF as a pivotal transcription regulator in diverse disease contexts and further emphasize the need to explore its role in MI‐induced cardiac remodeling.

It is unclear whether NKRF is involved in regulating CF function. This study investigated the effect of NKRF on cardiac remodeling post‐MI to deduce the underlying mechanism. The findings in this study establish NKRF as a therapeutic target for pathological cardiac remodeling and dysfunction post‐MI.

## 2 Results

### 2.1 NKRF Expression is Downregulated in Pathological Cardiac Remodeling

**Figure 1:**
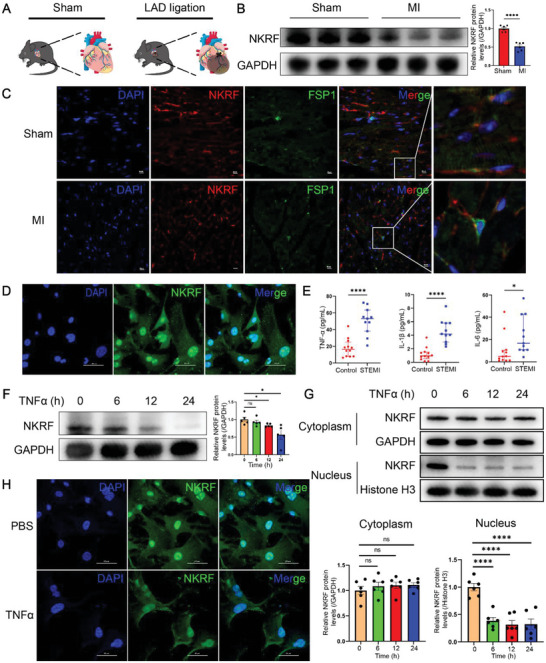
Downregulation of NKRF expression in cardiac fibroblasts (CFs) of the myocardial infarction (MI) border zone. A–C) Male C57BL/6J mice (aged 8 weeks) were subjected to MI by ligation of the left anterior descending coronary artery and euthanized at 28 days post‐MI. A) MI in mice. B) Western blotting and quantification of NKRF from the protein extracted within the MI border region in mice (n = 6). C) Immunofluorescence co‐staining of NKRF (red) and FSP1 (green) in the border zone of MI in mice (scale bar = 20 µm). D) Immunofluorescence staining shows the location of NKRF (green) in primary CFs (scale bar = 100 µm). E) Serum TNF‐α, IL‐1β, and IL‐6 levels in patients with ST‐segment elevation MI and normal healthy individuals. F–H) Primary CFs were treated with TNF‐α (10 ng mL^−1^) at the indicated time points. F) Western blotting and quantification of NKRF (n = 5). G) Immunoblotting and quantification of NKRF from isolated nuclear and cytoplasmic subcellular fractions of CFs (n = 6). H) Immunofluorescence staining of NKRF (green) in CFs treated with TNF‐α for 24 h (scale bar = 100 µm). Data are expressed as the mean ± SEM. NS, non‐significant, *p<0.05, and ****p<0.0001 (unpaired two‐tailed Student's t‐test). LAD, left anterior descending coronary artery; NKRF, NF‐κB‐repressing factor; FSP1, fibroblast‐specific protein 1.

We initially established a male C57BL/6J mouse (aged 8 weeks) model of MI based on a previous report to investigate the mechanism of pathological cardiac remodeling in a clinically relevant model (**Figure** **1**A).^[^ ^9c^ ^]^ NKRF expression significantly decreased in the MI border region after 4 weeks of MI (Figure 1B). We isolated CF, CM, and macrophage populations from ischemic mouse hearts following established protocols to investigate the role of NKRF in different cardiac cell types post‐MI.^[^ ^17^ ^]^ Interestingly, NKRF expression was significantly lower in CFs from the MI group than in those from the sham group, while no significant difference was observed in CMs and macrophages (Figure S1, Supporting Information).

Immunofluorescence staining showed that NKRF expression was significantly downregulated in CFs in the border region of MI (Figure 1C). Hence, we directed our focus towards understanding the specific role of NKRF in CFs during post‐MI cardiac remodeling. Osmotic pumps with Ang II were subcutaneously implanted into C57BL/6J mice (aged 8 weeks) to further elucidate the role of NKRF in CFs in pathological cardiac remodeling for 4 weeks to produce a pathological cardiac remodeling and fibrosis model of hypertension (Figure S2A, Supporting Information).^[^ ^18^ ^]^ Picrosirius red (PSR) staining showed that the ECM of the myocardium had a significant increase in the collagen level in hypertensive mice (Figure S2B, Supporting Information). Furthermore, NKRF expression in CFs decreased in hypertensive mice (Figure S2C, Supporting Information). These results suggest that NKRF expression is downregulated in CFs and plays an important role in orchestrating pathological cardiac remodeling and fibrosis.

Previous research illustrates that NKRF is predominantly detectable in the nucleoli, but some proteins are also present in the nucleoplasm and cytoplasm in C243 cell lines.^[^ ^19^ ^]^ However, the location of NKRF in primary CFs remains unknown. This point was addressed by isolating primary CFs from neonatal C57BL/6J mice (1–3 days old) as previously reported.^[^ ^20^ ^]^ Immunofluorescence staining showed that most of the isolated primary CFs expressed FSP1 (the CF marker protein). This indicated the specificity of the isolated CFs (Figure S3A, Supporting Information). Furthermore, immunofluorescence staining indicated that almost all isolated primary CFs expressed vimentin (another CF marker) without any expression of CD31 (endothelial cell marker) or cTnI (CM marker) (Figure S3B, Supporting Information). Moreover, NKRF was predominantly expressed in the nucleus (mainly in the nucleoli) in primary CFs, whereas some expression was detected in the cytoplasm using immunofluorescence staining (Figure 1D). This corroborated the findings in C243 cell lines.^[^ ^19^ ^]^

Many inflammatory factors are released during the acute inflammatory response; these inflammatory factors have a significant impact on the later stage of the repair and proliferative phase.^[^ ^3c^ ^]^ We measured serum TNF‐α, IL‐1β, and IL‐6 levels in patients with ST‐segment elevation myocardial infarction (STEMI) within 12 h from the onset of symptoms. Detailed demographic and clinical information about the patients and healthy donors is presented in Table S1 (Supporting Information). The levels of TNF‐α, IL‐1β, and IL‐6 significantly increased in patients with STEMI compared with those in healthy control individuals (Figure 1E). Furthermore, a significant elevation of TNF‐α mRNA levels was observed in the infarct border zone of the MI group compared with those in the sham group in C57BL/6J mice at 3 days post‐MI (Figure S4, Supporting Information). TNF‐α (10 ng mL^−1^), IL‐1β (10 ng mL^−1^), and IL‐6 (20 ng mL^−1^) were used to simulate the inflammatory environment in vitro to elucidate the effect of inflammatory factors on NKRF in CFs. Western blotting demonstrated that TNF‐α induced an earlier and gradual downregulation of NKRF expression at 12 h (Figure 1F), highlighting its crucial role. Meanwhile, no such effect was observed with IL‐1β (Figure S5A, Supporting Information). IL‐6 induced a significant decrease in NKRF expression at 24 h (Figure S5B, Supporting Information). Additionally, western blot analysis of isolated nuclear and cytoplasmic subcellular fractions of CFs confirmed that the reduction in NKRF expression was mainly reflected in the nucleus, but no significant changes were observed in the cytoplasm of CFs under TNF‐α induction (Figure 1G). Immunofluorescence staining further verified this finding (Figure 1H). In summary, these results demonstrated a significant downregulation of NKRF expression in pathological cardiac remodeling. Additionally, we identified the predominant nuclear localization of NKRF in primary CFs and its downregulation in response to TNF‐α induction in vitro. These findings underscore the potential significance of NKRF in orchestrating pathological cardiac remodeling and fibrosis, particularly involving CFs.

### 2.2 NKRF Protects against Cardiac Remodeling and Dysfunction Post‐MI

**Figure 2:**
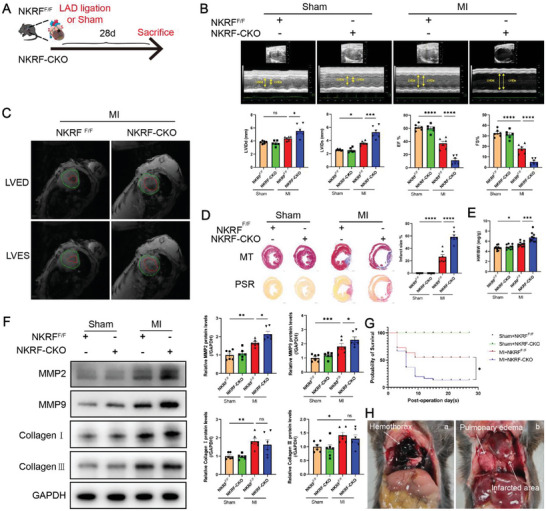
NKRF protects against cardiac remodeling and dysfunction post‐MI. A) Schematic diagram depicting the time course of MI‐induced cardiac remodeling and dysfunction in NKRF^F/F^ and NKRF‐CKO mice. B) Echocardiography and measured LVIDd, LVIDs, EF%, and FS% in NKRF^F/F^ and NKRF‐CKO mice (n = 6). C) Cardiac magnetic resonance (CMR) imaging at the left ventricular end‐diastolic phase (LVED) and end‐systolic phase (LVES) in NKRF^F/F^ and NKRF‐CKO mice. D) Masson's Trichrome (MT) and Picrosirius Red (PSR) staining from transverse cross‐sections of hearts obtained from NKRF^F/F^ and NKRF‐CKO mice, along with infarct size quantification (scale bar = 1000 µm, n = 7). E) Ratio of heart weight to body weight (HW/BW) of mice post‐MI 28 days (n = 10). F) Immunoblot and quantification of MMP2, MMP9, Collagen I, and Collagen III from the MI border region protein (n = 6). G) Kaplan–Meier survival analysis of NKRF‐CKO (n = 40) and NKRF^F/F^ mice (n = 20) after MI or sham operation. H) Deceased mice post‐MI; a. illustrates the hemothorax caused by cardiac rupture and b. shows the development of pulmonary edema. Data are expressed as the mean ± SEM. NS, non‐significant, *p<0.05, **p<0.01, ***p<0.001, and ****p<0.0001 by two‐way analysis of variance (ANOVA) with a Bonferroni multiple comparison test B,D,E,F) and log‐rank test G). NKRF^F/F^ and NKRF‐CKO, NKRF^flox/flox^ and NKRF^flox/flox^:Cre^S100a4^ mice; LAD, left anterior descending coronary artery; MI, myocardial infarction; LVIDd, left ventricular internal diastolic dimension; LVIDs, left ventricular internal systolic dimension; EF, left ventricular ejection fraction; FS, fractional shortening; MMP2, matrix metalloproteinase 2; MMP9, matrix metalloproteinase 9.

Next, we generated male CF‐specific NKRF‐knockout (NKRF^flox/flox^:Cre^S100a4^ [NKRF‐CKO]) mice to investigate the functional importance of CF NKRF in MI‐induced cardiac remodeling.^[^ ^21^ ^]^ NKRF‐CKO mice and littermates (NKRF^flox/flox^ [NKRF^F/F^]) were subjected to MI (**Figure** **2**A). These mice had specifically depleted NKRF protein in CFs but not in CMs and other tissues (Figure S6, Supporting Information). NKRF‐CKO mice exhibited lower cardiac function (left ventricular ejection fraction and fractional shortening, LVEF and FS, respectively) and larger left ventricular internal diastolic dimension (LVIDd) and systolic dimension (LVIDs) than NKRF^F/F^ mice post‐MI (Figure 2B). Similar findings were observed in cardiac magnetic resonance imaging (Figure 2C). NKRF‐CKO mice showed an increased infarct size after MI (Figure 2D). NKRF‐CKO mice had an increased heart weight/body weight (HW/BW) ratio compared with NKRF^F/F^ mice (Figure 2E). We analyzed the expression of MMP2, MMP9, collagen I, and collagen III in the border region where MI significantly induced the expression of these proteins to explore the changes in fibrosis‐associated proteins. MMP2 and MMP9 expression further increased in NKRF‐CKO mice compared with that in NKRF^F/F^ mice; however, collagen I and collagen III did not significantly change at the protein level (Figure 2F; Figure S7, Supporting Information). Correspondingly, the survival rate of the mice tended to be lower in NKRF‐CKO mice (35%, 26 deaths per 40 mice) than in NKRF^F/F^ mice (75%, 5 deaths per 20 mice) at 28 days post‐MI (P = 0.03; Figure 2G). Furthermore, nearly all deaths occurred within 10 days post‐MI, and most deaths occurred within 7 days after the procedure. Autopsies on all deceased mice indicated that hemothorax caused by cardiac rupture occurred in 2 of 5 deaths (40%) in NKRF^F/F^ mice and 19 of 26 deaths (73.1%) in NKRF‐CKO mice (P = 0.30, Figure 2H.a). The remaining deceased mice developed pulmonary edema; thus, we hypothesized that the cause of death was circulatory malfunction owing to heart failure (Figure 2H.b). In conclusion, CF‐specific NKRF knockout in mice exacerbates post‐MI cardiac remodeling, resulting in impaired cardiac function, an increased infarct size, and a decreased survival rate. These findings suggest that NKRF plays a protective role against cardiac remodeling and dysfunction post‐MI.

### 2.3 NKRF Inhibits CF Migration and Invasion by Downregulating MMP2 and MMP9 Expression

**Figure 3:**
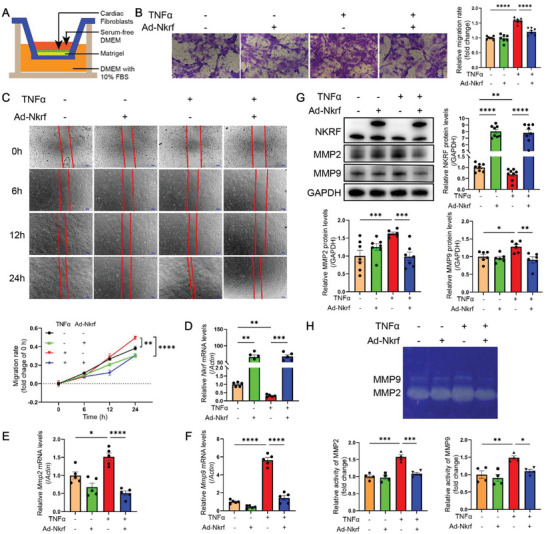
NKRF inhibits CF migration and invasion by downregulating MMP2 and MMP9 expression. A) Transwell invasion assay. B–H) Primary CFs transfected with Ad‐*Nkrf* or Ad‐Vector for 24 h were treated with TNF‐α (10 ng mL^−1^) for another 24 h. B) Transwell invasion staining and relative migration rate calculation (scale bar = 50 µm, n = 7). C) Wound healing assays and migration rate quantification at the indicated time points (scale bar = 200 µm, n = 7). D–F) Relative mRNA expression of *Nkrf*, *Mmp2*, and *Mmp9*, respectively (n = 5). G) Western blotting and quantification of NKRF (n = 8), MMP2 (n = 7), and MMP9 (n = 6). H) Gelatin zymography and quantitative analysis of MMP2 and MMP9 activities in cultured supernatants (n = 4). Data are the mean ± SEM. *p<0.05, **p<0.01, ***p<0.001, and ****p<0.0001 (two‐way ANOVA with Bonferroni multiple comparisons test). Ad‐*Nkrf*, NKRF adenovirus.

The upper compartment of the Transwell invasion system's polycarbonate membrane was coated with a layer of Matrigel to simulate the ECM in vitro. This ECM mimicry necessitates CFs to secrete MMPs to degrade the ECM and facilitate their migration into the lower compartment (**Figure** **3**A). TNF‐α significantly increased CF invasion toward the lower compartment, but this effect was inhibited by NKRF overexpression (Figure 3B). Similarly, a wound healing assay showed that TNF‐α significantly enhanced CF migration over time, and this increment was inhibited by NKRF overexpression (Figure 3C).

Increased MMP2 and MMP9 expression promotes increased CF migration.^[^ ^22^ ^]^ Inactivation of MMP9 and MMP2 in vivo prevents cardiac rupture post‐MI in mice and alleviates collagen accumulation, left ventricular dilatation, and cardiac dysfunction.^[^ ^23^ ^]^ Real time polymerase chain reaction (RT‐PCR) showed that NKRF mRNA levels significantly decreased after TNF‐α induction (Figure 3D). The mRNA levels of MMP2 and MMP9 significantly increased after TNF‐α induction, and this effect was significantly inhibited by NKRF overexpression (Figure 3E,F). The changes at the protein level were consistent with those at the mRNA level (Figure 3G). Gelatin in‐gel zymography showed that TNF‐α significantly increased the activities of MMP2 and MMP9, but NKRF significantly inhibited this increase (Figure 3H). Furthermore, three small interfering RNAs (siRNAs) of NKRF were constructed for reverse knockdown verification; the third siRNA showed the highest knockdown efficiency in CFs (Figure S8A and S8B, Supporting Information). The decrease in TNF‐α‐induced NKRF mRNA levels in the knockdown experiment was consistent with that observed in the overexpression assay (Figure S8C, Supporting Information). TNF‐α‐induced increases in MMP2 and MMP9 levels were further enhanced following NKRF knockdown at the mRNA level (Figure S8D and S8E, Supporting Information). The changes at the protein level were consistent with those at the mRNA level (Figure S8F, Supporting Information). NKRF knockdown significantly amplified the TNF‐α‐induced increase in MMP2 and MMP9 activities (Figure S8G, Supporting Information). In summary, NKRF overexpression inhibits CF migration and invasion by downregulating MMP2 and MMP9 expression and activities. This suggests a potential role of NKRF in regulating ECM remodeling and CF behavior in the context of pathological cardiac remodeling.

### 2.4 NKRF Inhibits the Stability of *Mmp2* and *Mmp9* mRNA via Inhibiting HuR Expression

**Figure 4:**
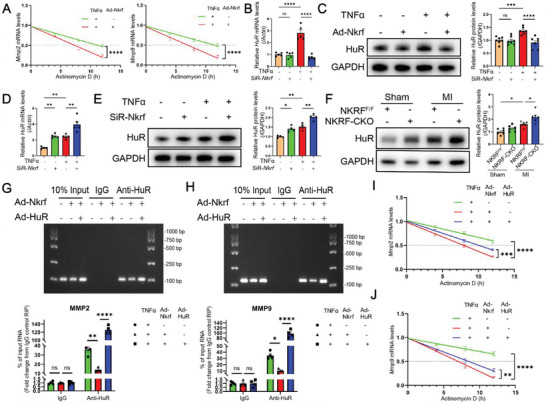
NKRF inhibits the stability of *Mmp2* and *Mmp9* mRNA by inhibiting HuR expression. A) NKRF inhibits mRNA stability of *Mmp2* (n = 6) and *Mmp9* (n = 6). Significant differences were observed at 12 h. (B,C) NKRF inhibits TNF‐α‐induced HuR expression in CFs at the mRNA (B, n = 5) and protein (C, n = 7) levels. (D,E) NKRF knockdown enhances TNF‐α‐induced HuR expression in CFs at the mRNA (D, n = 5) and protein levels (E, n = 4). F) Western blotting and quantification of HuR protein extracted from the MI border region in NKRF^F/F^ mice and NKRF‐CKO mice (n = 6). (G,H) Agarose gel electrophoresis and RT‐PCR results of *Mmp2* (G, n = 4) and *Mmp9* (H, n = 4) mRNA enriched by the HuR antibody in RIP experiments. (I,J) HuR rescues the NKRF‐inhibited mRNA stability of *Mmp2* (I, n = 4) and *Mmp9* (J, n = 4). Significant differences were observed at 12 h. Data are the mean ± SEM. NS, non‐significant, *p<0.05, **p<0.01, ***p<0.001, and ****p<0.0001 by unpaired two‐tailed Student's t‐test (A), two‐way ANOVA with Bonferroni multiple comparisons test B–F), and one‐way ANOVA with Bonferroni multiple comparisons test G–J). Ad‐*Nkrf*, NKRF‐overexpressing adenovirus; Ad‐*HuR*, HuR‐overexpressing adenovirus; SiR‐*Nkrf*, *Nkrf* siRNA (small interfering RNA); NKRF^F/F^, NKRF^flox/flox^ mice; NKRF‐CKO, NKRF^flox/flox^:Cre^S100a4^ mice; MI, myocardial infarction; Anti‐HuR, HuR antibody.

NKRF regulated MMP2 and MMP9 at the mRNA and protein levels. This suggests that NKRF is involved in the regulation of MMP2 and MMP9 at the transcriptional or post‐transcriptional level. NKRF can play a role by binding to an NRE (AATTCCTCTGA) in the promoters of *IFN‐β* and *IL‐8* to inhibit their transcription.^[^ ^10^, ^11^ ^]^ However, we did not find NRE sequences in the promoters of *Mmp2* and *Mmp9* in NCBI Gene. We designed primers covering the gene promoter regions of *Mmp2* and *Mmp9* for chromatin immunoprecipitation (ChIP) experiments to confirm whether NKRF exerts transcriptional negative regulation by directly binding to the gene promoters (Figure S9A, Supporting Information). Unfortunately, NKRF did not enrich any fragments of the *Mmp2* (Figure S9B, Supporting Information) and *Mmp9* (Figure S9C, Supporting Information) gene promoter regions. Subsequently, we blocked mRNA synthesis by actinomycin D to observe the remaining mRNA levels of *Mmp2* and *Mmp9* at different time points in CFs activated by TNF‐α. The remaining mRNA levels in all groups were gradually downregulated after actinomycin D treatment. The remaining mRNA levels of *Mmp2* and *Mmp9* were significantly decreased by NKRF overexpression upon treatment with actinomycin D at 12 h (**Figure** **4**A). The above results suggest that NKRF regulates MMP2 and MMP9 at the post‐transcriptional level by inhibiting their mRNA stability.

HuR (also known as embryonic lethal abnormal vision‐like 1, ELAVL1) is a ubiquitous RNA‐binding protein that regulates gene expression through the post‐transcriptional pathway.^[^ ^24^ ^]^ It plays a role in various pathological mechanisms by affecting the stability of *TGF‐β*, *P53*, and *MMP9* mRNAs.^[^ ^25^ ^]^ Moreover, we previously found that HuR binds adenylate uridylate‐rich elements of *Mmp2* and *Mmp9* mRNAs at the 3′ untranslated region (3′ UTR), resulting in increased *Mmp2* and *Mmp9* mRNA stability.^[^ ^26^ ^]^ We examined whether NKRF regulated HuR expression in CFs to verify whether NKRF regulates MMP2 and MMP9 expression via HuR. TNF‐α significantly increased HuR transcription (Figure 4B) and translation (Figure 4C) according to RT‐PCR and western blotting assays. This was significantly inhibited by NKRF overexpression, but no effect on HuR was observed at the baseline. In contrast, TNF‐α‐induced HuR transcription (Figure 4D) and translation (Figure 4E) were further increased when NKRF was knocked down. Moreover, NKRF knockdown significantly increased HuR transcription (Figure 4D) and translation (Figure 4E) in the absence of TNF‐α administration. MI significantly induced HuR expression at the MI border region in NKRF^F/F^ mice and further increased HuR expression in NKRF‐CKO mice (Figure 4F). Further, RNA immunoprecipitation (RIP) experiments showed that the levels of *Mmp2* (Figure 4G) and *Mmp9* (Figure 4H) mRNAs enriched by the HuR antibody were significantly reduced by NKRF overexpression; however, this was reversed with HuR overexpression. The results obtained via RT‐PCR were consistent with those obtained using agarose gel electrophoresis. The stability of *Mmp2* (Figure 4I) and *Mmp9* (Figure 4J) mRNAs inhibited by NKRF was rescued by HuR overexpression after actinomycin D blocked mRNA synthesis. In summary, NKRF inhibits the stability of *Mmp2* and *Mmp9* mRNAs by downregulating HuR expression. This provides insights into the post‐transcriptional regulatory mechanism of NKRF in modulating MMP2 and MMP9 expression during cardiac remodeling.

### 2.5 NKRF Inhibits HuR Transcription by Binding the *HuR* Promoter via an NF‐κB‐Dependent Mechanism

**Figure 5:**
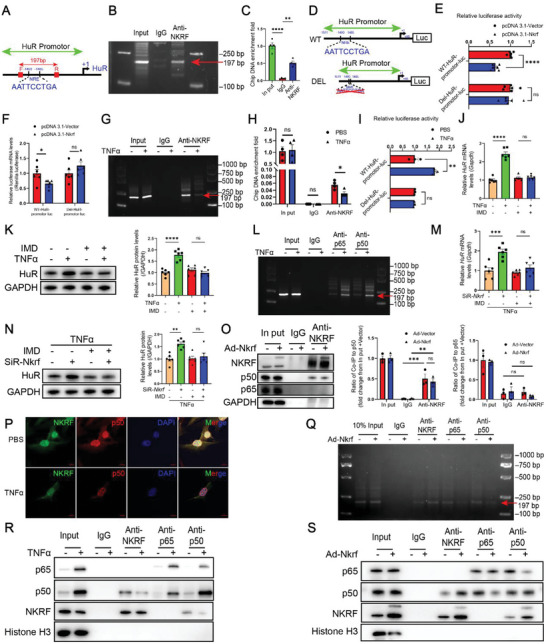
NKRF inhibits HuR transcription by binding to the *HuR* promoter via an NF‐κB dependent mechanism. A) Negative regulatory element (NRE) in the *HuR* promoter region. B,C) Agarose gel electrophoresis B) and RT‐PCR results (C, n = 4) using NRE in the *HuR* promoter region enriched with NKRF antibody as a template in chromatin immunoprecipitation (ChIP) experiments. D) Firefly luciferase expression plasmid construction with the wild‐type *HuR* promoter (WT, pGL3‐WT‐HuR promoter) and *HuR* promoter with deleted NRE sequence (DEL, pGL3‐DEL‐HuR promoter). E) A dual luciferase reporter (DLR) assay was used to analyze the effect of NKRF on firefly luciferase activity in HEK293T cells (n = 6). F) Relative luciferase mRNA expression levels in HEK293T cells (n = 6). G,H) Agarose gel electrophoresis G) and RT‐PCR results (H, n = 4) using NRE in the *HuR* promoter region enriched with NKRF antibody as a template in ChIP experiments. I) The DLR assay was used to analyze the effect of TNF‐α on firefly luciferase activity in CFs (n = 3). J,K) The NF‐κB pathway is required for TNF‐α‐induced HuR expression at the mRNA (J, n = 6) and protein levels (K, n = 6) in CFs. L) Agarose gel electrophoresis using NRE in the *HuR* promoter region enriched with p65 and p50 antibodies as a template in ChIP experiments. M,N) The NF‐κB pathway is required for the transcriptional regulation of HuR by NKRF at the mRNA (M, n = 6) and protein levels (N, n = 6) in CFs. O) Immunoblotting analysis of NKRF, p65, and p50 in co‐immunoprecipitation (Co‐IP) experiments (n = 3). P) Immunofluorescence staining of NKRF (green) and p50 (red) in CFs treated with TNF‐α (10 ng mL^−1^) or PBS for 24 h (scale bar = 10 µm). Q) Agarose gel electrophoresis using NRE in the *HuR* promoter region enriched with NKRF, p65, and p50 antibodies as a template in ChIP experiments. R,S) Immunoblotting analysis of NKRF, p65, and p50 in Co‐IP experiments. R) Primary CFs were treated with TNF‐α (10 ng mL^−1^) or PBS for 24 h. S) Primary CFs were transfected with Ad‐*Nkrf* or Ad‐Vector for 48 h following treatment with TNF‐α (10 ng mL^−1^) for 24 h. Data are the mean ± SEM. NS, non‐significant, *p<0.05, **p<0.01, ***p<0.001, and ****p<0.0001 by one‐way ANOVA with Bonferroni multiple comparisons test (C,O), two‐way ANOVA with Bonferroni multiple comparisons test (J,K,M,N), and unpaired two‐tailed Student's t‐test (E,F,H,I). NRE, negative regulatory element; Anti‐NKRF, NKRF antibody; Luc, luciferase; WT, pGL3‐WT‐HuR promoter plasmid; DEL, pGL3‐DEL‐HuR promoter plasmid; pcDNA3.1‐NKRF, NKRF overexpression plasmid; pcDNA3.1‐Vector, vector control plasmid; IMD, NF‐κB pathway inhibitor IMD 0354; SiR‐*Nkrf*, *Nkrf* small interfering RNA (siRNA); Ad‐*Nkrf*, NKRF overexpression adenovirus; Ad‐Vector, vector control adenovirus; DAPI, 4′,6‐diamidino‐2‐phenylindole; Anti‐p65, p65 antibody; Anti‐p50, p50 antibody.

We found similar NRE sequences (AATTCCTGA) at −1493 to −1485 sites upstream of the transcription start site in the *HuR* promoter. We designed forward and reverse primers with an interval length of 197 bp across the predicted NRE sequence for ChIP experiments to verify whether NKRF binds to the NRE sequence in the *HuR* promoter (**Figure** **5**A). ChIP showed that the NKRF antibody significantly enriched a band of 197 bp in length, whereas no band was found at the same location in the negative control IgG lane in CFs (Figure 5B). RT‐PCR assays showed similar results using pull‐down DNA fragments as the template (Figure 5C). The role of NKRF in regulating HuR transcription was verified by subcloning the wild‐type (WT) HuR promoter and HuR promoter with deleted (DEL) NRE sequence into the firefly luciferase expression vector pGL3‐Basic to perform a dual‐luciferase reporter (DLR) assay in HEK293T cells (Figure 5D). NKRF significantly inhibited the activity of firefly luciferase in the WT group, but this difference was not observed in the DEL group (Figure 5E). The expression of firefly luciferase mRNA was consistent with the DLR findings according to RT‐PCR assays (Figure 5F). ChIP agarose gel electrophoresis revealed that the enrichment of NKRF to the predicted NRE sequence was downregulated in CFs during TNF‐α treatment (Figure 5G). The RT‐PCR results of DNA fragments pulled down by NKRF antibody or IgG as templates were in agreement with ChIP agarose gel electrophoresis results (Figure 5H). The constructed WT and DEL luciferase reporter plasmids were transfected into CFs, and DLR demonstrated that TNF‐α significantly promoted the activity of firefly luciferase in the WT group but not in the DEL group (Figure 5I).

The canonical NF‐κB pathway is typified by the inducible phosphorylation and degradation of IκBα resulting from TNF‐α ligation of TNFR1. This further releases the established p65:p50 heterodimer complex into the nucleus, resulting in the transcription of downstream target genes.^[^ ^27^ ^]^ NF‐κB activates the transcription of HuR in human gastric cancer cell lines.^[^ ^28^ ^]^ We employed the inhibitor IMD 0354 to investigate involvement of the NF‐κB pathway in HuR transcription in primary CFs. This inhibitor blocks IκB kinase, leading to the inhibition of IκBα phosphorylation and degradation, resulting in cytoplasmic localization of p65:p50 dimers.^[^ ^29^ ^]^ TNF‐α induced HuR expression at the mRNA and protein levels in the control group; however, this effect disappeared after IMD 0354 pretreatment (Figure 5J,K). This suggested that the NF‐κB pathway mediates TNF‐α‐induced HuR expression at the transcriptional level. ChIP assays showed that p65 and p50 bound to the *HuR* promoter, and this binding was significantly enhanced after TNF‐α treatment (Figure 5L). Furthermore, TNF‐α‐induced enrichment of p65 and p50 to the *HuR* promoter was inhibited after blocking the NF‐κB pathway by IMD 0354; however, this did not affect the downregulation trend of NKRF enrichment to the *HuR* promoter (Figure S10, Supporting Information). These results suggest that the NF‐κB pathway mediates the transcriptional regulation of HuR induced by TNF‐α in CFs through p65 and p50 binding to the *HuR* promoter. Next, we investigated the impact of NKRF on HuR expression by inhibiting the NF‐κB pathway. Knockdown of NKRF significantly elevated HuR expression at the mRNA and protein levels in TNF‐α‐treated CFs. However, this effect was not observed when CFs were pretreated with IMD 0354 (Figure 5M,N). This result suggests that the negative transcriptional regulation of HuR by NKRF requires the NF‐κB pathway. Our investigation of the negative transcriptional regulation by NKRF through the NF‐κB pathway revealed interaction of NKRF with p50 (but not p65) in total CFs according to co‐immunoprecipitation (Co‐IP) results (Figure 5O). Immunofluorescence staining confirmed co‐localization of NKRF with p50 (Figure 5P, top). ChIP analysis demonstrated that NKRF overexpression hindered p65 and p50 binding to the *HuR* promoter in TNF‐α‐treated CFs (Figure 5Q). Additionally, Co‐IP (Figure 5R) and immunofluorescence staining (Figure 5P) results demonstrated a significant reduction in NKRF binding to p50 upon TNF‐α‐induced NF‐κB activation. Reverse pull‐down experiments with p65 and p50 antibodies showed a notable increase in p65‐p50 heterodimer complex formation following TNF‐α treatment (Figure 5R). Moreover, Co‐IP revealed that NKRF overexpression increased NKRF binding with p50, while p50‐p65 binding in the nucleus decreased in TNF‐α‐treated CFs (Figure 5S). In summary, TNF‐α treatment leads to NKRF expression downregulation in the nucleus. This promotes p50 binding to p65 to form the p65:p50 heterodimer complex. This complex subsequently binds to the *HuR* promoter and promotes its transcription.

### 2.6 HuR Reverses the Inhibitory Effect of NKRF on CF Migration and Invasion by Upregulating MMP2 and MMP9 Expression

**Figure 6:**
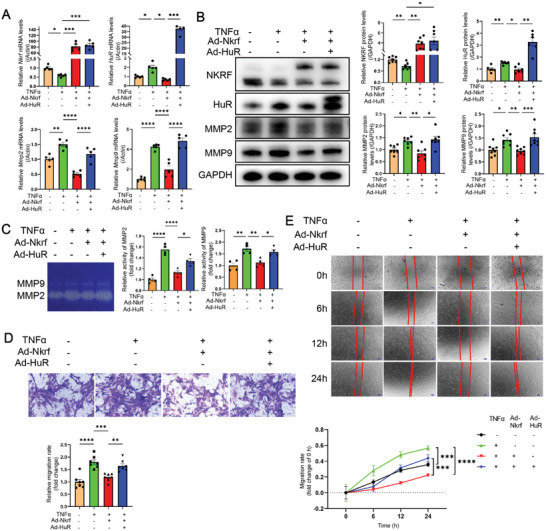
HuR reverses the inhibitory effect of NKRF on CF migration and invasion by upregulating MMP2 and MMP9. A,B) HuR reversed the inhibitory effect of NKRF on TNF‐α‐induced expression of MMP2 and MMP9 at the mRNA (A, n = 5) and protein (B, n = 6 at least) levels in CFs. C) Gelatin zymography showed that HuR reversed the inhibitory effect of NKRF on TNF‐α‐induced enhanced activity of MMP2 and MMP9 in cultured CFs supernatants. D) Transwell invasion staining and relative migration rate calculation (scale bar = 50 µm, n = 7). E) Wound healing assays and migration rate quantification at the indicated time points (scale bar = 200 µm, n = 7). Data are the mean ± SEM. *p<0.05, **p<0.01, ***p<0.001, and ****p<0.0001 by one‐way ANOVA with Bonferroni multiple comparisons test. Ad‐*Nkrf*, NKRF‐overexpressing adenovirus; Ad‐*HuR*, HuR‐overexpressing adenovirus.

Subsequently, we sought to investigate whether HuR could counteract the inhibitory effect of NKRF on MMP2 and MMP9 expression at the mRNA and protein levels. We observed a significant reversal of NKRF‐mediated inhibition on *Mmp2* and *Mmp9* mRNA levels through adenoviral overexpression of HuR in addition to NKRF (**Figure** **6**A). Moreover, the trend at the protein level was consistent with that at the mRNA level (Figure 6B). Concurrently, the third siRNA was identified as the most effective siRNA among the three candidates for HuR knockdown (Figure S11A and S11B, Supporting Information). Subsequent reverse verification demonstrated that HuR knockdown significantly counteracted the promoting effect of NKRF knockdown on MMP2 and MMP9 expression at the mRNA and protein levels (Figure S11C and S11D, Supporting Information). Gelatin in‐gel zymography revealed that HuR substantially restored the inhibitory effect of NKRF on MMP2 and MMP9 activity (Figure 6C). Consistent findings were observed in the HuR knockdown rescue experiment (Figure S11E, Supporting Information). The Transwell invasion experiment demonstrated that HuR overexpression significantly counteracted the inhibitory effect of NKRF on CF invasion and promoted CF mobility (Figure 6D). Similarly, the wound healing assay illustrated that HuR significantly reversed the inhibitory effect of NKRF on CF migration over time (Figure 6E). In summary, HuR upregulates MMP2 and MMP9 expression, and its overexpression reverses the inhibitory effect of NKRF on CF migration and invasion. This highlights the critical role of HuR in modulating the regulatory function of NKRF in CFs during pathological cardiac remodeling.

### 2.7 HuR Knockdown Protects against Deteriorating Cardiac Remodeling and Dysfunction Post‐MI in NKRF‐CKO Mice

**Figure 7:**
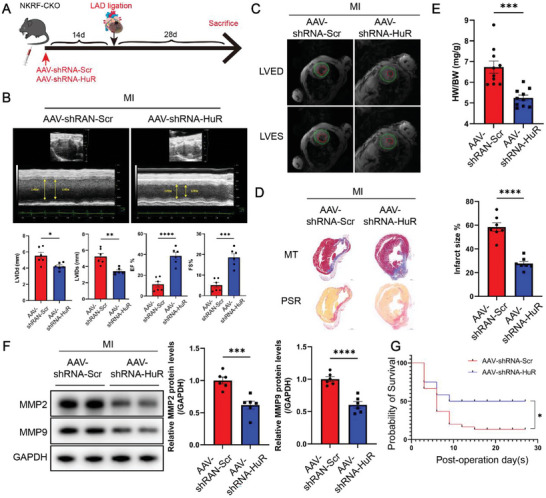
HuR knockdown protects against deteriorating cardiac remodeling and dysfunction post‐MI in NKRF‐CKO mice. A) Schematic diagram depicting the time course of MI‐induced cardiac remodeling and dysfunction in NKRF‐CKO mice receiving AAV‐shRNA‐*HuR* or AAV‐shRNA‐Scr. B) Echocardiography and measured LVIDd, LVIDs, EF%, and FS% in NKRF‐CKO mice (n = 6). C) Cardiac magnetic resonance imaging at the left ventricular end‐diastolic phase (LVED) and end‐systolic phase (LVES) in NKRF‐CKO mice. D) Masson's trichrome (MT) and picrosirius red (PSR) staining from transverse cross‐sections of heart tissues obtained from NKRF‐CKO mice (scale bar = 1000 µm, n = 7). E) Ratio of heart weight to body weight (HW/BW) in NKRF‐CKO mice 28 days post‐MI (n = 10). F) Immunoblotting and quantification of MMP2 and MMP9 in the MI border region in NKRF‐CKO mice (n = 6). G) Kaplan–Meier survival analysis of NKRF‐CKO mice treated with AAV‐shRNA‐Scr (n = 40) and AAV‐shRNA‐*HuR* (n = 19) post‐MI. Data are the mean ± SEM. *p<0.05, **p<0.01, ***p<0.001, and ****p<0.0001 by unpaired two‐tailed Student's t‐test (B,D,E,F) and log‐rank test G). NKRF‐CKO mice, NKRF^flox/flox^:Cre^S100a4^ mice; MI, myocardial infarction; LAD, left anterior descending coronary artery; AAV‐shRNA‐*HuR*, adeno‐associated virus short hairpin RNA‐*HuR*; AAV‐shRNA‐Scr, adeno‐associated virus short hairpin RNA‐scramble control; CMR, cardiac magnetic resonance; LVIDd, left ventricular internal diastolic dimension; LVIDs, left ventricular internal systolic dimension; LVEF, left ventricular ejection fraction; FS, fractional shortening.

An adeno‐associated virus (AAV) carrying short hairpin RNA targeting HuR (AAV‐shRNA‐*HuR*) driven by the *FSP1* promoter in CFs was developed to investigate if HuR knockdown ameliorates the deteriorating cardiac function in NKRF‐CKO mice in vivo. Tail vein injection of AAV‐shRNA‐*HuR* was performed 14 days prior to simulating MI in NKRF‐CKO mice (**Figure** **7**A). Western blotting verified the knockdown efficiency of HuR in CFs isolated from two NKRF‐CKO mice before modeling (Figure S12, Supporting Information). Echocardiography revealed that HuR knockdown in NKRF‐CKO mice significantly improved cardiac function, as evidenced by reduced LVIDd and LVIDs, along with increased LVEF and FS at 28 days post‐MI (Figure 7B). Similar results were demonstrated via in vivo cardiac magnetic resonance imaging of the left ventricular short axis (Figure 7C). HuR knockdown minimized the infarct size (Figure 7D) and the HW/BW ratio (Figure 7e) in NKRF‐CKO mice. HuR knockdown significantly inhibited MMP2 and MMP9 mRNA and protein expression in the MI border region (Figure 7F; Figure S13, Supporting Information). HuR knockdown partially restored the survival rate post‐MI (68%, 6 deaths per 19 mice versus 35%, 26 deaths per 40 mice, P = 0.04) in NKRF‐CKO mice (Figure 7G). In conclusion, HuR knockdown protects against deteriorating cardiac remodeling and dysfunction post‐MI in NKRF‐CKO mice. This suggests a potential therapeutic strategy to mitigate the adverse effects of NKRF deficiency on cardiac function.

### 2.8 NKRF is a Potential Therapeutic Target to Inhibit Cardiac Remodeling and Dysfunction via HuR Post‐MI

**Figure 8:**
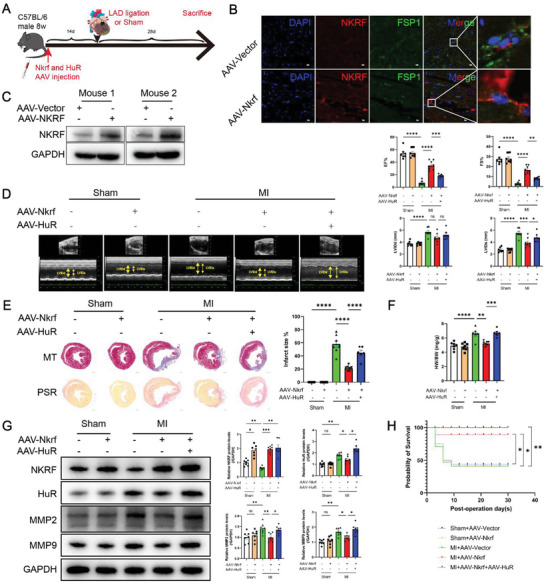
HuR reverses the protection effects of NKRF on cardiac remodeling and dysfunction post‐MI in vivo. A) Schematic diagram depicting the time course of MI‐induced cardiac remodeling and dysfunction in C57BL/6J mice receiving AAV‐*Nkrf*/AAV‐Vector (NKRF) and AAV‐*HuR*/AAV‐Vector (HuR). (B,C) Immunofluorescence staining and immunoblot analysis show that NKRF (red) was significantly overexpressed and co‐localized with the FSP1 (green) in transverse cross‐sections of hearts B) and overexpressed in isolated CFs C) after treatment with AAV‐*Nkrf* for 14 days. D–F) Echocardiography and measured LVIDd, LVIDs, EF%, and FS% (D, n = 7), MT and PSR staining from transverse cross‐sections of hearts (E, scale bar = 1000 µm, n = 7), and ratio of heart weight to body weight (HW/BW) (F, n = 7) in C57BL/6J mice treated with AAV‐*Nkrf* and AAV‐*HuR* 28 days post‐MI. G) Immunoblot and quantification of NKRF, HuR, MMP2, and MMP9 from the MI border region protein (n = 7). H) Kaplan–Meier survival analysis in C57BL/6J mice treated with AAV‐*Nkrf* and AAV‐*HuR* after MI. Data are mean ± SEM. NS, non‐significant, *p<0.05, **p<0.01, ***p<0.001, and ****p<0.0001 by one‐way ANOVA with Bonferroni multiple comparisons test (D,E,F,G) and log‐rank test H). AAV‐*Nkrf* and AAV‐Vector (NKRF), adeno‐associated virus NKRF and control; AAV‐*HuR* and AAV‐Vector(HuR), adeno‐associated virus HuR and control; DAPI, 4′,6‐diamidino‐2‐phenylindole; FSP1, Fibroblast‐specific protein 1; LAD, left anterior descending coronary; MI, myocardial infarction; LVIDd, left ventricular internal diastolic dimension; LVIDs, left ventricular internal systolic dimension; EF, left ventricular ejection fraction; FS, fractional shortening; MT, Masson's Trichrome staining; PSR, Picrosirius Red staining.

AAV‐*Nkrf* injections were administered to C57BL/6J mice, followed by MI surgery 14 days later to underscore the therapeutic potential of NKRF in post‐MI cardiac remodeling. AAV‐*Nkrf* was specifically expressed using the *FSP1* promoter in CFs (**Figure** **8**A). This provided valuable insights into the targeted modulation of cardiac repair by NKRF post‐MI. Immunofluorescence staining (Figure 8B) and western blotting (Figure 8C) revealed a significant and specific increase in NKRF expression in CFs prior to the operation. Echocardiography demonstrated that NKRF overexpression significantly improved MI‐induced deterioration of cardiac function at 28 days post‐MI (Figure 8D). NKRF also protected against MI‐induced enlarged infarct size (Figure 8E) and increased HW/BW ratio (Figure 8F). Furthermore, NKRF inhibited protein expression of MMP2 and MMP9 in the MI border region (Figure 8G). In addition, NKRF overexpression significantly improved the survival rate of mice post‐MI (93%, 1 death per 15 mice versus 62%, 10 deaths per 26 mice, P = 0.03; Figure 8H).

We also injected AAV‐*HuR* (specifically expressed in CFs) in mice that received AAV‐*Nkrf* to clarify whether HuR mediated the protective effect of NKRF post‐MI. HuR overexpression significantly reversed NKRF‐improved cardiac function (Figure 8D), infarct size (Figure 8E), HW/BW ratio (Figure 8F), MMP2 and MMP9 expression in the MI border region (Figure 8G), and survival rate (63%, 9 deaths per 24 mice versus 93%, 1 death per 15 mice, P = 0.04; Figure 8H). In summary, NKRF emerges as a potential therapeutic target to inhibit cardiac remodeling and dysfunction post‐MI, and its protective effects are mediated (at least in part) through HuR regulation. This suggests a promising avenue for cardiac remodeling intervention.

## 3 Discussion

Left ventricular remodeling is characterized by cardiac fibrosis. It is a common consequence of various cardiovascular diseases that contribute to heart failure and increased cardiovascular mortality.^[^ ^1^ ^]^ Despite its significance, the precise mechanism governing this pathological process remains elusive. Here, we provided new insights into the role of NKRF in CFs during cardiac remodeling following MI. We found that NKRF protects against cardiac remodeling and increases the survival rate of mice post‐MI in vivo. NKRF inhibited MMP2 and MMP9 expression in CFs and decreased the invasion and migration of CFs in vitro. Mechanistically, NKRF inhibited the transcriptional expression of HuR via an NF‐κB‐dependent manner, thus decreasing the stability of *Mmp2* and *Mmp9* mRNAs.

NKRF is a transcriptional silencer of specific NF‐κB‐targeting genes, including *IFN‐β*, *IL‐8*, *IP‐10*, and *iNOS* via the NRE in their promoters.^[^ ^11^, ^12^, ^13^, ^30^ ^]^ Most of the available knowledge regarding NKRF was established using cell lines in vitro; however, the role of NKRF in human diseases is poorly understood. NKRF is constitutively expressed in all tested human tissues; this implies a possible role of NKRF in the pathophysiology of human diseases.^[^ ^11a^ ^]^ NKRF expression is reduced in PBMCs of patients with stable chronic obstructive pulmonary disease, and decreased NKRF expression potentially increases chronic systemic inflammation by promoting IL‐8 transcription.^[^ ^12^ ^]^ This study also found decreased NKRF expression in pathological fibrosis myocardium. The adult mammalian myocardium is rich in CFs that play a vital role in maintaining the ECM network.^[^ ^31^ ^]^ We further demonstrated that NKRF expression was inhibited in CFs post‐MI and decreased in hypertensive mouse CFs. These results further corroborated the role of NKRF in pathological cardiac remodeling. TGF‐β has been extensively used as a pro‐fibrotic growth factor to induce CFs.^[^ ^9^, ^32^ ^]^ CFs are exposed to many inflammatory factors induced by damage‐associated molecular patterns in the acute inflammatory phase post‐MI. Prolonged CF exposure to inflammatory cytokines prevents premature acquisition of a synthetic myofibroblast phenotype and promotes a matrix‐degrading phenotype characterized by MMP synthesis.^[^ ^3^, ^33^ ^]^ This study found that TNF‐α significantly induced NKRF expression downregulation over time in CFs; this mainly occurred in the nucleus. These in vitro findings were consistent with in vivo observations. These findings suggest that NKRF plays a role in cardiac remodeling post‐MI in CFs. However, previous studies found that NKRF expression is significantly upregulated in THP1 cells treated with heated *Mycobacterium tuberculosis* and in PBMCs derived from patients with tuberculosis.^[^ ^13^ ^]^ The differential expression of NKRF in different cells in response to different stimuli suggests that NKRF plays its role through different mechanisms. In this study, NKRF expression was downregulated in hypertensive fibrosis myocardial CFs; therefore, NKRF may also have a developmental effect on hypertensive cardiac remodeling. However, one of the limitations of the current study is that it cannot be extended to elucidate cardiac remodeling in the hypertensive mouse model. Hence, future studies are needed to uncover the regulatory effects of NKRF on hypertensive cardiac remodeling.

We found that NKRF deficiency in CFs significantly deteriorated late cardiac function and increased the infarct size and heart weight in vivo. NKRF‐CKO mice showed low postoperative survival. Postmortem examination showed that deceased NKRF‐CKO mice post‐MI tended to have a higher incidence of hemothorax caused by cardiac rupture than NKRF^F/F^ mice, although the difference was not statistically significant. The hallmarks of the proliferative phase of cardiac repair are the expansion of CF population and conversion into synthetic myofibroblast phenotypes.^[^ ^34^ ^]^ These changes are vital to maintain a structurally stable ECM to prevent cardiac rupture.^[^ ^1^, ^35^ ^]^ We observed an increase in the mortality trend of cardiac rupture in NKRF‐CKO mice, urging us to determine changes in ECM synthetic phenotype marker proteins and ECM‐degrading enzymes in the infarct border region. We found that NKRF knockout in CFs did not affect the expression of ECM synthetic phenotype proteins collagen I and collagen III, whereas the expression of ECM‐degrading enzymes MMP2 and MMP9 significantly increased. This may explain why NKRF‐CKO mice had increased mortality and an increased incidence of cardiac rupture. MMP2 and MMP9 deficiency in vivo protects against cardiac rupture and improves left ventricular enlargement, fibrosis, and dysfunction post‐MI;^[^ ^23^, ^36^ ^]^ our findings are consistent with previous results.^[^ ^23^, ^25^, ^36^ ^]^ Furthermore, we built a Transwell invasion system in vitro that mimics the ECM in vivo. NKRF significantly inhibited the invasion of CFs induced by inflammatory cytokines; moreover, a wound healing assay provided similar results. CFs are the principle cell type responsible for cardiac fibrosis; they secrete a variety of MMPs to promote ECM degradation, thus increasing CF migration, fibrosis, and negative cardiac remodeling.^[^ ^37^ ^]^ Increased MMP2 and MMP9 expression contributes to CF migration in vitro.^[^ ^22^, ^38^ ^]^ Consistently, we found that NKRF significantly inhibited the increase in inflammatory cytokine‐induced expression and activities of MMP2 and MMP9. Therefore, we preliminarily concluded that NKRF prevents cardiac remodeling post‐MI by inhibiting MMP2 and MMP9 expression in CFs.

HuR (ELAVL1) is a ubiquitously expressed member of the Hu/ELAV family of RNA‐binding proteins and is one of the best‐studied regulators of cytoplasmic mRNA fate.^[^ ^39^ ^]^ Mechanistically, HuR acts to increase target mRNA stability by binding to adenylate uridylate‐rich elements in the 3ʹ UTR via its RNA recognition motifs.^[^ ^39^, ^40^ ^]^ HuR expression in the injured heart significantly increased post‐MI, and HuR was expressed in CFs rather than in CMs in the hearts of adult mice.^[^ ^25^, ^41^ ^]^ HuR expression was enhanced in diabetic mouse hearts, and exosomes derived from HuR‐deficient macrophages significantly inhibited the expression of fibrogenesis genes *Tgf‐β*, *Mmp9*, *Col1α1*, and *Col3α1* in CFs.^[^ ^42^ ^]^ HuR plays a role in a variety of pathophysiological processes by binding to the 3ʹ UTR of *Mmp9* mRNA to increase its stability.^[^ ^43^ ^]^ Furthermore, we previously showed that HuR plays a protective role against abdominal aortic aneurysm by increasing mRNA stability via binding to the 3ʹ UTR of *Mmp2* and *Mmp9* mRNAs.^[^ ^26^ ^]^ This study verified that NKRF significantly inhibited HuR expression in vivo and in vitro. This further attenuates its binding to *Mmp2* and *Mmp9* mRNAs, which reduces the stability of these mRNAs and downregulates their expression. The current findings confirmed those of previous reports.^[^ ^26^, ^43^ ^]^ Additionally, we found that HuR significantly reversed the inhibitory effect of NKRF on CF migration and invasion in vitro and destroyed the protective effects of NKRF on cardiac function, infarct size, and survival rate in vivo. Our data are consistent with the previously reported negative effects of elevated HuR expression after MI.^[^ ^25a^ ^]^

NKRF exerts negative transcriptional regulation through binding to the NRE region in the promoters of target genes.^[^ ^10^, ^11^ ^]^ We found a similar sequence of the NRE in the promoter of *HuR* and hypothesized that the negative regulation of NKRF on *HuR* is through binding to the promoter NRE of HuR; this hypothesis was verified using ChIP and DLR assay. NKRF downregulation in the CF nucleus induced by TNF‐α attenuated its binding to the *HuR* promoter NRE, thereby promoting HuR expression. These experiments indicated that NKRF plays a negative transcriptional regulatory role on HuR. NKRF plays a transcriptional repressive role in human airway smooth muscle cells treated with TNF‐α.^[^ ^10^ ^]^ However, NKRF plays a dual role in the transcription of target genes: NKRF plays a transcriptional negative role in regulating the *IL‐8* promoter in the absence of IL‐1β induction, whereas NKRF plays a transcriptional positive role in the presence of IL‐1β.^[^ ^11b^ ^]^ This suggests that the transcriptional regulatory role of NKRF is affected by other transcription factors. Additionally, HuR transcription is positively controlled via the NF‐κB pathway.^[^ ^28^ ^]^ We further verified that HuR transcription induced by TNF‐α was mediated by the NF‐κB pathway via the heterodimer complex p65:p50 binding to the NRE in the *HuR* promoter. NKRF and the p65:p50 heterodimer complex bound to the NRE in the promoter of HuR and played negative and positive roles in transcriptional regulation, respectively. Therefore, the interaction between NKRF and the p65:p50 heterodimer complex is worth investigating. We found that NF‐κB is required for the transcriptional negative regulation of HuR by NKRF. Mechanistically, NKRF inhibited formation of the p65:p50 heterodimer complex through competitive binding with p50. After TNF‐α induction, NKRF expression downregulation in the CF nucleus attenuated this competition and promoted binding of the p65:p50 heterodimer complex to the NRE of the *HuR* promoter to induce its transcription. The negative transcriptional regulatory effect of NKRF is realized through a reduction in the number of binding events with pro‐transcription factors, rather than the reduction in binding force. This mechanism of NKRF action was previously reported, wherein NKRF expression was downregulated, thus increasing RNA polymerase II occupancy at the *IL‐8* promoter to enhance IL‐8 transcription in PBMCs of patients with severe chronic obstructive pulmonary disease.^[^ ^12^ ^]^

These results suggest that NKRF plays a negative regulatory role in HuR transcription that ensues when NKRF is overexpressed in the TNF‐α‐induced inflammatory background. In contrast, NKRF overexpression does not enhance the negative transcriptional regulatory role in the absence of TNF‐α; instead, NKRF knockdown reduces its regulatory effect. We speculate that the underlying mechanism is the saturation of NKRF and p50 binding in the absence of TNF‐α, and excessive NKRF cannot bind to p50. Thus, NKRF expression downregulation releases competitive binding with p50 in the presence of TNF‐α. This finding suggests that NKRF may be a therapeutic target to protect against left ventricular remodeling post‐MI while reducing side effects in non‐inflammatory tissues. Recent studies have shed light on the role of NKRF in fibrotic conditions in different organs. MiR‐29b regulates NKRF expression, impacts mesangial cell proliferation, the release of inflammatory mediators, and interstitial fibrosis in Henoch Schönlein purpura nephritis.^[^ ^44^ ^]^ Furthermore, miR‐802 directly binds to the 3ʹ UTR of NKRF in the context of obesity‐related nephropathy. This influences fibrosis and inflammatory responses in the kidneys.^[^ ^45^ ^]^ These findings from other fibrotic diseases corroborate the potential significance of NKRF as a key player in fibrosis regulation across different tissues. Cardiac repair post‐MI involves a complex series of events finely orchestrated by several cells, including macrophages, neutrophils, CMs, CFs, and endothelial cells.^[^ ^3c^ ^]^ This study investigated the effects of NKRF in CFs on cardiac remodeling post‐MI but did not observe it in other cell types. Indeed, exploring the role of NKRF in cardiac remodeling in other cell types may hold significant value to further understand the comprehensive regulatory mechanisms in post‐MI cardiac repair.

## 4 Conclusion

NKRF serves as a transcriptional silencer in CFs and protects against cardiac remodeling post‐MI. Mechanistically, NKRF inhibits the transcription of HuR by binding to the NRE within the *HuR* promoter in an NF‐κB‐dependent manner, thus inhibiting the stability of *Mmp2* and *Mmp9* mRNAs. Our findings suggest that early NKRF targeting in patients post‐MI is an effective strategy to protect against late cardiac remodeling. These findings provide new insights into the treatment of cardiac remodeling post‐MI.

## 5 Experimental Section

### Animals

The Cre/LoxP system was used to generate cardiac fibroblast (CF)‐specific NKRF knockout (NKRF‐CKO) mice. Briefly, NKRF‐CKO mice were bred by crossing the NKRF^flox/flox^ strain (Shanghai Model Organisms Center, Inc., Shanghai, China) with the S100a4‐Cre strain (Stock No. 012641; Jackson Laboratories). Littermates (NKRF^flox/flox^ [NKRF^F/F^]) were used as controls. Eight‐week‐old male NKRF‐CKO and NKRF^F/F^ mice were used in the present study. Eight‐week‐old male C57BL/6J mice were purchased from the Beijing Vital River Laboratory Animal Technology (Beijing, China). All experiments and groups were blinded by digitally encoding mice. The serotype 9 adeno‐associated viruses (AAVs) specifically encoding NKRF (AAV‐*Nkrf*), HuR (AAV‐*HuR*), and short hairpin RNA‐HuR (AAV‐shRNA‐*HuR*, 5ʹ‐GCACAGAGATTCAGGTTCT‐3ʹ) in CFs were purchased from WZ Biosciences Inc. Jinan, China. The transfection efficiency of AAVs (2.8×10^11^ vg mouse^−1^ of each AAV) was verified after 14 days of caudal intravenous injection.

These 8‐week‐old male C57BL/6J, NKRF^F/F^, and NKRF‐CKO mice underwent left anterior descending (LAD) coronary ligation, as previously described.^[^ ^9b^ ^]^ Briefly, the LAD was ligated with a 7‐0 wire at 2–3 mm from the distal part of the left atrial appendage under 2% isoflurane anesthesia. The sham mice were subjected to a similar procedure, sans ligation. The mouse model of hypertension received subcutaneously implanted osmotic pumps (Alzet Model 2004, CA, USA) into mice to deliver saline or angiotensin II (400 ng kg^−1^ min^−1^; Cat. No. HY‐13948; Monmouth Junction, NJ, USA) for 4 weeks. The mice were euthanized by intraperitoneal injection of sodium pentobarbital (50 mg kg^−1^) at the end of 4‐weeks modeling, and their blood samples and hearts were harvested, while their body and heart weight were measured. The blood samples were collected from the left ventricular cavity using heparin as an anticoagulant. Serum was obtained by centrifuging blood samples at 1,000 × *g* for 15 min at 4 °C and stored at −80 °C. Histological studies involved rinsing the heart with phosphate‐buffered saline (PBS), fixing it with 4% formaldehyde, and embedding it in paraffin. The heart was snap‐frozen with liquid nitrogen and stored at −80 °C for molecular biochemistry studies. Prior to conducting this study, the cardiac functional changes was initially explored, infarct size, and heart weight/body weight (HW/BW) ratio in S100a4‐Cre and NKRF^F/F^ mice following MI. No significant differences were observed upon comparing the post‐MI cardiac parameters between these two groups (Figure S14, Supporting Information). Hence, NKRF^F/F^ mice were utilized as the control group. The Institutional Animal Care and Use Committee at Qilu Hospital of Shandong University (DWLL‐2020‐082) approved all animal protocols in the study. All animal experiments were conducted in accordance with the National Institutes of Health Guide for the Care and Use of Laboratory Animals. All efforts were made to minimize animal suffering.

### Echocardiography

The cardiac structure and function of the mice were assessed using transthoracic echocardiography (VisualSonic VeVo 2100 Imaging System, Toronto, Canada) at the end of 4 weeks of modeling. The mice were anesthetized with 2% isoflurane inhalation, placed on a heated platform maintained at 37±1 °C, and connected with an electrocardiograph (ECG). The left ventricular internal diastolic dimension (LVIDd) and left ventricular internal systolic dimension (LVIDs) were recorded using M‐mode echocardiography in the parasternal long‐axis view. The left ventricular ejection fraction and fractional shortening were automatically calculated.

### Mouse Cardiac Magnetic Resonance (CMR) Imaging

Mouse CMR imaging was performed as previously reported.^[^ ^9f^ ^]^ A 4.7 T MRI system (BioSpec 47/40; Bruker, Ettlingen, Germany) with an ECG and respiratory gating (SA Instruments, Stony Brook, NY, USA) was used to perform CMR imaging in vivo. Mice were anesthetized with 2% isoflurane and placed on a heated platform to maintain the body temperature at 37±1 °C. Needle electrodes were fixed on the fore and hind limbs to obtain ECG signals, and an R‐wave was used to generate trigger pulses for image acquisition. T1W‐CMR black‐blood imaging with a FLASH cine sequence was performed in the axial direction to cover the left ventricle. The imaging parameters were as follows: field of view (FOV) = 25 × 25 mm^2^, matrix size = 192 × 192, slice thickness = 1.0 mm, number of slices = 6, TR = 38.3 ms, TE = 2.8 ms, flip angle = 15°, number of averages = 5, total scan time = 7 min 21 s.

### Histology, Immunofluorescence, Masson's trichrome (MT), and Picrosirius Red (PSR) Staining

Sections between the suture and apex of the heart were used as the infarct area. The infarct border area was defined as the interface area between the infarct and non‐infarct areas on the short‐axis section. The heart tissues (1 mm below the ligation) were cut into 4‐µm‐thick transverse sections along the horizontal long axis using a microtome (RM2235; Leica Microsystems, Inc., Mannheim, Germany). Serial sectioning was used for MT and PSR staining to evaluate myocardial fibrosis and collagen deposition, respectively. Infarct size was calculated as the infarct circumference divided by the total left ventricular circumference in the entire visual field of the section.

Immunofluorescence staining of NKRF and FSP1 involved dewaxing the sections, followed by antigen repair (Cat. No. C1034; Solarbio, Beijing, China) and treatment with 0.1% Triton X‐100 (Cat. No. GC204003; Servicebio, Wuhan, China) in PBS for 10 min. The sections were incubated with 2.5% normal goat serum (Cat. No. G1208; Servicebio) in PBS for 30 min at room temperature (23–27 °C) and treated with antibodies against NKRF (Cat. No. sc‐365568; Santa Cruz, TX, USA) and FSP1 (Cat. No. 16105‐1‐AP; Proteintech, Wuhan, China) overnight at 4 °C. The sections were subsequently washed three times with PBS and incubated with Alexa Fluor 594 (Cat. No. ab150120; Abcam, Cambridge, MA, USA) and Alexa Fluor 488 (Cat. No. ab150081; Abcam) secondary antibodies (1:200) for 1 h in the dark at 37 °C. The nuclei were labeled with 4′,6‐diamidino‐2‐phenylindole (DAPI, Cat. No. ab104139; Abcam). Immunofluorescence staining of the infarct border areas was visualized using a Zeiss 73447 confocal laser scanning microscope (Oberkochen, Germany). Representative images were randomly selected from each group.

Immunofluorescence staining involved fixing primary CFs with 4% formaldehyde, permeabilizing with 0.1% Triton X‐100 (Cat. No. GC204003; Servicebio), blocking with 2.5% normal goat serum (Cat. No. G1208; Servicebio) in PBS for 30 min at room temperature (23–27 °C), and treating with antibodies against NKRF (Cat. No. ab168829; Abcam), p50 (Cat. No. 66992‐1‐Ig; Proteintech), FSP1 (Cat. No. 16105‐1‐AP; Proteintech), vimentin (Cat. No. ab92547; Abcam), CD31 (Cat. No. ab28364; Abcam), and cTnI (Cat. No. ab47003; Abcam) overnight at 4 °C, followed by incubation with fluorescent secondary antibodies, DAPI staining, and confocal microscopy as described above. Representative images were randomly selected from each group.

### Cytokine Measurement

11 initial patients were consecutively recruited with ST‐segment elevation myocardial infarction within 12 h of symptom onset at Qilu Hospital of Shandong University. A control group of 12 age‐ and sex‐matched healthy volunteers were enrolled. All participants signed informed consent forms, and the study was approved by the Institutional Ethical Committee of Qilu Hospital of Shandong University (Approval No. 2021–151) and was conducted in agreement with Helsinki Declaration principles. Human serum samples were collected and stored at ‐80°C. Enzyme‐linked immunofluorescent assay kits were used according to the manufacturer's instructions to determine TNF‐α (Cat. No. DTA00D; RD Systems, Minneapolis, MN, USA), IL1B (Cat. No. DLB50; RD Systems), and IL6 (Cat. No. D6050; RD Systems) levels.

### Reagents and Antibodies

Recombinant murine TNF‐α protein (Cat. No. 315‐01A) was purchased from PEPROTECH (Rocky Hill, NJ, USA). IMD 0354 was purchased from Selleck (Shanghai, China). Additional reagents and antibodies were mentioned in the specific methods.

### Isolation of Primary Cardiac Fibroblasts (CFs) and Cell Culture and Transfection

Primary neonatal mouse CFs were isolated from mice 1–3 days post‐birth as previously described.^[^ ^9d^ ^]^ Briefly, mice were anesthetized with isoflurane (0.5%) and cleaned with 70% ethanol, and the heart was removed. The ventricular tissue was cut into 1 mm^3^ small pieces and digested with D‐Hank's solution containing 0.0125% Collagenase II (Cat. No. LS004176; Worthington, Lakewood, NJ, USA) at 4 °C (spinning 50 rpm) overnight. The supernatant was discarded on the following day, and the tissue was further digested with D‐Hank's solution containing 0.0125% pancreatin without ethylenediaminetetraacetic acid (EDTA) (Gibco, Grand Island, NY, USA) in a 37 °C water bath at a low spinning speed for 2 min. The cell suspension was collected and filtered through a 100‐µm polypropylene cell strainer (Cat. No 15–1100; Biologix, Jinan, China) into an equal volume of Dulbecco's modified Eagle's medium (DMEM; Gibco BRL, Gaithersburg, MD, USA) containing 10% fetal bovine serum (FBS) (Cat. No. 10100147C; Thermo Fisher Scientific, Waltham, MA, USA) to terminate the digestion. This procedure was repeated until all tissues were digested. The sample was centrifuged at 1,000 rpm for 5 min, the supernatant was discarded, and the cell precipitate was resuspended in DMEM containing 10% FBS, 1% penicillin, and streptomycin (Cat. No. 10378016; Thermo Fisher Scientific) prior to incubation at 37°C with 5% CO_2_ for 1.5 h (Thermo Model 371, Marietta, OH, USA). The cells that adhered to the dishes were CFs.

CFs were transfected with small interfering RNA (siRNA) using Lipofectamine RNAi MAX Transfection Reagent (Cat. No. 13778150, Thermo Fisher Scientific) according to the manufacturer's instructions. Commercial synthesis of siRNAs against NKRF, HuR, and the scramble control was performed by Ribobio (Guangzhou, Guangdong, China). The target sequences for the siRNAs against NKRF and HuR were as follows: SiR‐*Nkrf*‐1, 5ʹ‐CCGGTTCCAAATTCCATGT‐3ʹ, SiR‐*Nkrf*‐2, 5ʹ‐CCAGCATGCCAAGAAACTT‐3ʹ; SiR‐*Nkrf*‐3, 5ʹ‐CCTGTAGCAACCAACATGT‐3ʹ; SiR‐*HuR*‐1, 5ʹ‐CCAAGAGGAACTACGAAGT‐3ʹ, SiR‐*HuR*‐2, 5ʹ‐CAAGCTCAGAGGTCATCAA‐3ʹ; SiR‐*HuR*‐3, 5ʹ‐GCACAGAGATTCAGGTTCT‐3ʹ.

Adenovirus Nkrf (Ad‐*Nkrf*) and HuR (Ad‐*HuR*) were obtained from WZ Biosciences Inc. Jinan, China. Ad‐*Nkrf* and Ad‐*HuR* were transfected according to the manufacturer's instructions. Transfection with adenovirus (MOI = 200) was performed in CFs for 48 h, followed by subsequent overexpression experiments. When cells reached 70% to 80% confluency, CFs (passages 1–3) were incubated in serum‐free DMEM overnight before treatment with TNF‐α (10 ng mL^−1^).

### Isolation of Distinct Cardiac Cell Populations from Infarcted Mouse Hearts

Isolation of distinct cardiac cell populations from infarcted mouse hearts was performed 4 weeks after MI, as previously described.^[^ ^17^ ^]^ Cardiomyocytes were isolated using established protocols.^[^ ^46^ ^]^ Hearts were dissociated into single‐cell suspensions using the Skeletal Muscle Dissociation Kit (Miltenyi Biotech, Shanghai, China) for CFs and macrophages. Macrophages were positively selected using Anti‐F4/80‐coated magnetic beads (Cat. No. 130‐110‐443; Miltenyi Biotech) from CFs following the manufacturer's instructions. Purified cells were then collected by centrifugation at 300 × g for 5 min at 4 °C for subsequent protein extraction.

### Protein Extraction and Western Blot Analysis

The harvested heart samples and CFs were lysed in radioimmunoprecipitation assay buffer (Sigma‐Aldrich, St Louis, MO, USA) with a 1X protease inhibitor cocktail (Cat. No. 04693132001; Roche, Indianapolis, IN, USA). The samples were fully lysed in a tissue homogenizer (FLUKO, Shanghai, China). The sample was centrifuged at 10000 × g for 10 min at 4 °C and the supernatant was extracted for protein quantification and denaturation. Separation of nuclear and cytoplasmic proteins was conducted via a commercial Minute™ Cytoplasmic & Nuclear Extraction Kit (Cat. No. SC‐003; INVENT, Plymouth, MN, USA) according to the manufacturer's instructions. The concentration of the extracted protein was measured using a BCA Protein Assay Kit (Thermo Fisher Scientific) and adjusted to a similar concentration using the extraction reagent. The extracted protein was separated using 4–10% gradient Bis–Tris SDS‐Gels (Bio‐Rad, Hercules, CA, USA) and transferred to nitrocellulose membranes (Millipore, Billerica, MA, USA). The membranes were incubated in 5% non‐fat milk at room temperature (23–27 °C) for 1 h followed by incubation with primary antibodies against NKRF (Cat. No. 14693‐1‐AP; Proteintech), HuR (Cat. No. 11910‐1‐AP; Proteintech), MMP2 (Cat. No. 10373‐2‐AP; Proteintech), MMP9 (Cat. No. 10375‐2‐AP; Proteintech), GAPDH (Cat. No. 2118; Cell Signaling Technology, Danvers, MA, USA), histone‐H3 (Cat. No. 17168‐1‐AP; Proteintech), collagen I (Cat. No. 66761‐1‐Ig; Proteintech), collagen III (Cat. No. 22734‐1‐AP; Proteintech), p65 (Cat. No. ET1603‐12; HUABIO, Hangzhou, China), and p50 (Cat. No. 66992‐1‐Ig; Proteintech) at 4 °C overnight. On the next day, the membranes were washed with Tris‐buffered saline and Tween 20 (TBST) three times and incubated with horseradish peroxidase (HRP)‐conjugated secondary antibody (Cat. No. ab6721 for rabbit; ab6728 for mouse; Abcam) at room temperature (23–27 °C) for 1 h. Thereafter, the membranes were washed with TBST three times, and protein bands were visualized (AMERSHAM ImageQuant 800, GE Healthcare Bio‐Sciences AB, Sweden) using an ECL western blotting detection kit (Millipore, Temecula, CA, USA). ImageJ (National Institutes of Health, Bethesda, MD, USA) was used to quantify the intensity of the bands. The protein expression levels were normalized to those of GAPDH or histone H3.

### Total RNA Isolation and Real‐Time Polymerase Chain Reaction (RT‐PCR) Analysis

TRIzol reagent (Invitrogen, Carlsbad, CA, USA) was used to extract total RNA from the cells according to the manufacturer's protocol. RNA (1 µg) was reverse‐transcribed to cDNA using a PrimeScript RT Reagent Kit (Takara Biomedical Technology). RT‐PCR amplification was performed using SYBR PCR mix (Roche, Mannheim, Germany) with specific primers (Table S2, Supporting Information) on a Bio‐Rad CFX96^TM^ Real‐Time PCR detection system (Bio‐Rad Laboratories Inc.). The real‐time PCR amplification procedure was conducted at 10 min at 95 °C, followed by 35 consecutive cycles of amplification (30 s at 95°C for denaturation, 30 s at 60 °C for annealing, and 30 s at 72 °C for extension). Actin RNA was used as the internal control. The ΔCt method was used to calculate expression levels, and the 2−ΔΔCt method was used for comparison.

### Cell Migration and Invasion Assay

The invasion mobility assay was performed using 24‐well Transwell Permeable Supports (Costar, Kennebunk, ME, USA) plates with polycarbonate membrane filters of 8‐µm pore size, as previously described.^[^ ^22^ ^]^ A layer of Matrigel (Corning, NY, USA) was applied on the upper compartment of the polycarbonate membrane of the Transwell invasion system to mimic the ECM in vitro. After treatment, CFs (70% to 80% confluency) were placed in the upper part of the chamber containing serum‐free DMEM and incubated for 24 h. A chemoattractant (10% FBS in DMEM) was loaded into the lower well of the system and incubated. CFs on the upper surface of the filter were removed using a cotton swab and those on the lower surface of the membrane were fixed with 4% formaldehyde and stained with crystal violet (Cat. No. G1014; Servicebio). Subsequently, the stained CFs were air‐dried for 30 min after washing out the additional stain with double‐distilled water. CFs mobilized to the lower surface of the membrane were counted using an inverted microscope (Olympus, Tokyo, Japan) under a light field.

CF migration was determined using wound healing assays. After treatment, a monolayer of CFs was cultured in 6‐well plates. After attachment to the plates, a 1‐mm‐wide tip was used to scrape along the well diameter to create the wound space. Complete DMEM (10% FBS) was used to wash off the shed cells. Then, the CFs were cultured in complete DMEM medium in an incubator at 37 °C with 5% CO_2_ for 0 h, 6 h, 12 h, and 24 h. Migration micrographs were obtained using an inverted microscope (Olympus) at different time points. Image J was employed to measure the wound distance. The average of three measurements in each image was used as the average distance. The migration rates were calculated as follows

(1) $$\begin{array}{ccc} & & \mathrm{Migration}\;\mathrm{rate}\;=\\ & & \frac{\left(\mathrm{average}\;\mathrm{distance}\;\mathrm{at}\;\mathrm{time}\;0\;h\right)-\left(\mathrm{average}\;\mathrm{distance}\;\mathrm{at}\;\mathrm{time}\;6\;h,\;12\;h,\;\mathrm{and}\;24\;h\right)}{\left(\mathrm{average}\;\mathrm{distance}\;\mathrm{at}\;\mathrm{time}\;0\;h\right)}\; \end{array}$$

### Gelatin Zymography

MMP2 and MMP9 activities were assessed using gelatin zymography. The supernatant of the treated CFs was collected, mixed with 5x loading dye, and separated using 8% sodium dodecyl sulfate‐polyacrylamide gel electrophoresis containing 0.1% gelatin at 120 V for 3 h. The gels were washed and shaken twice in a 2.5% Triton X‐100 solution for 30 min and incubated in 50 mL reaction buffer (40 mM Tris‐HCl, pH 8.0, 10 mM CaCl_2_, 0.01% NaN_3_) at 37°C for 12 h. Then, the gels were stained with 0.25% Coomassie brilliant blue R‐250 (Cat. No. ST031; Beyotime, Shanghai, China) in 50% methanol and 10% acetic acid for 1 h. MMP9 and MMP2 showed clear bands after destaining (10% acetic acid, 20% methanol) twice for 30 min.

### RNA Immunoprecipitation (RIP) Assay and mRNA Stability Experiments

The RIP assay was performed using the Magna RIP kit (Cat. No. 17–701; Millipore), according to the manufacturer's instructions. Briefly, whole‐cell lysates were incubated with 5 µg rabbit IgG (Cat. No. 2729; Cell Signaling Technology) or HuR antibody (Cat. No. 11910‐1‐AP; Proteintech) at 4 °C overnight, whereby antibodies were precoated with magnetic protein A/G beads (Cat. No. CS203178; Millipore). The pulled‐down protein–RNA complex was washed and incubated with proteinase K buffer (30 min at 55 °C; Cat. No. CS203218; Millipore). Phenol:chloroform:isoamyl alcohol (125:24:1, Solarbio) was used to extract RNA, and a PrimeScript RT Reagent Kit (Takara Biomedical Technology) was used to synthesize cDNA. The product was subjected to PCR, agarose gel electrophoresis, and RT‐PCR using the SYBR PCR mix (Bio‐Rad).

*Mmp2* and *Mmp9* mRNA stability assays were performed in primary CFs treated with TNF‐α (10 ng mL^−1^). Cardiac fibroblasts were cultured in fresh DMEM containing Actinomycin D (5 µg mL^−1^; Cat. No. 129935; Sigma‐Aldrich) following TNF‐α treatment (10 ng mL^−1^). The mRNA was extracted from these CFs at 0 h, 4 h, 8 h, and 12 h followed by reverse transcription and RT‐PCR.

### Chromatin Immunoprecipitation (ChIP) Assay

The Magna ChIP^TM^ HiSens Chromatin Immunoprecipitation Kit (Cat. No. 0025; Millipore) was used to perform the ChIP assay according to the manufacturer's recommendations. Briefly, ≈2 × 10^6^ primary CFs cultured in a 10 cm petri dish were used per immunoprecipitation sample. CFs were fixed for 10 min at room temperature (23–27 °C) and sonicated on a Bioruptor UCD‐200TM‐EX for 16 cycles of 30 s ON and 30 s OFF at high power. DNA bound to NKRF, p65, and p50 was precipitated using anti‐NKRF (Cat. No. 14693‐1‐AP; Proteintech), anti‐p65 (Cat. No. ET1603‐12; HUABIO, Hangzhou, China), and anti‐p50 (Cat. No. 14220‐1‐AP; Proteintech). Rabbit IgG (Cat. No. 2729; Cell Signaling Technology) was used as a control. The precipitated DNA was subjected to PCR following agarose gel electrophoresis and RT‐PCR to calculate the fold enrichment of ChIP DNA using specific HuR promoter primers. These primer sequences were listed in Table S2 (Supporting Information).

### Dual‐Luciferase Reporter (DLR) Assay

Wild‐type *HuR* promoter (pGL3‐WT‐HuR promoter) and deleted negative regulatory element (NRE) sequence *HuR* promoter (pGL3‐DEL‐HuR promoter) were subcloned into the firefly luciferase expression vector pGL3‐Basic plasmid to perform DLR assays using the DLR Assay System (Cat. No. E1910; Promega, Madison, WI, USA) in HEK293T cells. HEK293T cells (Cat. No. CRL‐11268, ATCC, Manassas, VA, USA) were cultured in DMEM supplemented with 10% FBS. A Renilla luciferase control reporter vector plasmid was used as an internal control to monitor transfection efficiencies as previously described.^[^ ^39b^ ^]^ Plasmids were transfected into HEK293T cells using Lipofectamine 3000 Transfection Reagent (Cat. No. L3000001; Thermo Fisher Scientific) according to the manufacturer's instructions. The relative luciferase activity was represented by the activity ratio of firefly luciferase to Renilla luciferase using a Centro XS^3^ LB 960 microplate luminometer (Berthold Technologies, Bad Wildbad, Germany) 48 h post‐transfection.

### Co‐Immunoprecipitation (Co‐IP) Analysis

For the Co‐IP assay, post‐treatment CFs were lysed in IP buffer (150 mM saline, 50 mM Tris‐HCl, 1% NP‐40, pH 7.8) containing mammalian cell‐specific protease inhibitor cocktail (Merck, Darmstadt, Germany). The concentration of the extracted protein was measured using a BCA Protein Assay Kit (Thermo Fisher Scientific) and adjusted to similar concentrations (across extracts) using the extraction reagent. Briefly, 1 mg protein was incubated with magnetic protein A/G beads (Cat. No. HY‐K0202; MedChemExpress, Shanghai, China) precoated with 5 µg of rabbit IgG (Cat. No. 2729; Cell Signaling Technology), anti‐NKRF (Cat. No. 14693‐1‐AP; Proteintech), anti‐p65 (Cat. No. ET1603‐12; HUABIO), or anti‐p50 (Cat. No. 14220‐1‐AP; Proteintech) antibody at 4 °C overnight with constant rotation. Then, the beads were washed five times with PBST (136.89 mM NaCl, 2.67 mM KCl, 8.1 mM Na_2_HPO_4_, 1.76 mM KH_2_PO_4_, and 0.5% Tween 20). The precipitated proteins were eluted from the magnetic beads using 2x loading buffer (Cat. No. P0015F; Beyotime) and boiled for 5 min. Immunoprecipitated proteins were subjected to western blot analysis.

### Statistical Analysis

All data were expressed as the mean ± standard error of the mean (SEM). All analyses were performed using GraphPad Prism 9 (GraphPad Software, San Diego, CA, USA). Each experiment was independently repeated at least three times to perform statistical analysis. All data were tested for normality using the Shapiro–Wilk normality test. Each group (P>0.05) in the normality test indicated that the data were approximately normally distributed. An unpaired two‐tailed Student's t‐test was used for the normally distributed data to determine statistically significant differences between two groups. One‐way analysis of variance (ANOVA) followed by Bonferroni multiple comparisons test (with a mixed model using different numbers of replicates per condition) was performed to determine the statistical difference between multiple groups with one variable and normal distribution. A two‐way ANOVA followed by the Bonferroni multiple comparisons test was used to compare multiple groups with more than one variable. Non‐normally distributed data were analyzed using the nonparametric statistical Kruskal–Wallis test followed by Dunn's post‐hoc test for multiple comparisons. A Kaplan–Meier curve was created to illustrate the cumulative survival post‐MI. The differences in cumulative survival were analyzed using a log‐rank test. Statistical significance was set at P<0.05 unless otherwise stated. The tests used to assess significance were detailed in the figure legends.

## Conflict of Interest

The authors declare no conflict of interest.

## Author Contributions

C.G. conceived the study, designed experiments, conducted experiments, analyzed and interpreted results, and wrote the manuscript. W.J., W.Y., Q.D., T.Z., Z.W., W.S., C.Z., F.Y., and W.Z. designed and conducted experiments and analyzed and interpreted results. B.X., X.Y., F.X., and Q.Z. helped with the design of the study experiments. C.Z., M.Z., and J.K. conceived the study, directed and supervised the research, and wrote the manuscript.

## Supporting information

Supporting Information

## Data Availability

The data that support the findings of this study are available from the corresponding author upon reasonable request.
