# Supplementary material for: NKRF in Cardiac Fibroblasts Protects against Cardiac Remodeling Post‐Myocardial Infarction via Human Antigen R
Source: Adv Sci (Weinh). 2023 Sep 5;10(30):2303283. doi: 10.1002/advs.202303283 (PMC10602562; doi:10.1002/advs.202303283)
Supplement: Supplementary file 1 — Supporting Information [file ADVS-10-2303283-s001.pdf]

ADVANCED SCIENCE
Open Access

Supporting Information

for *Adv. Sci.*, DOI 10.1002/advs.202303283

NKRF in Cardiac Fibroblasts Protects against Cardiac Remodeling Post-Myocardial Infarction via Human Antigen R

Chenghu Guo, Wei Ji, Wei Yang, Qiming Deng, Tengfei Zheng, Zunzhe Wang, Wenhai Sui, Chungang Zhai, Fangpu Yu, Bo Xi, Xiao Yu, Feng Xu, Qunye Zhang, Wencheng Zhang, Jing Kong, Meng Zhang* and Cheng Zhang**

Supporting Information

NKRF in Cardiac Fibroblasts Protects Against Cardiac Remodeling Post-Myocardial Infarction via Human Antigen R

Chenghu Guo, Wei Ji, Wei Yang, Qiming Deng, Tengfei Zheng, Zunzhe Wang, Wenhai Sui, Chungang Zhai, Fangpu Yu, Bo Xi, Xiao Yu, Feng Xu, Qunye Zhang, Wencheng Zhang, Jing Kong, Meng Zhang,* Cheng Zhang**

Supplemental figures and figure legends

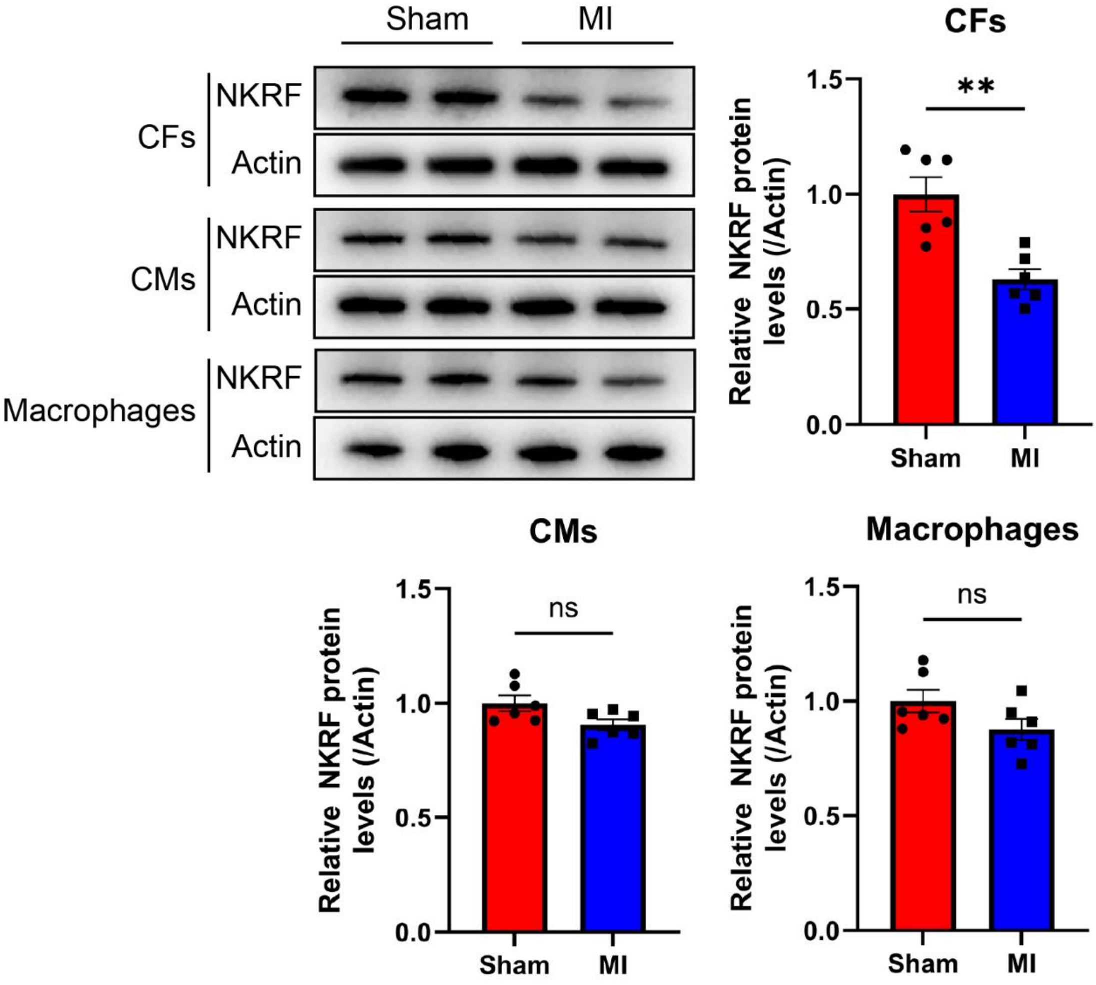

Figure S1. NKRF expression in different cardiac cell types post-myocardial infarction (MI).

Isolation of cardiac fibroblasts (CFs), cardiomyocytes (CMs), and macrophages from ischemic mouse hearts revealed a significant downregulation of NKRF expression in CFs (n = 6) in the MI group compared with that in the sham group, while no significant difference was observed in CMs (n = 6) and macrophages (n = 6). Data are the mean ± SEM. P-values were determined using unpaired two-tailed Student's t-test. NS, non-significant, *P<0.05, and **P<0.01.

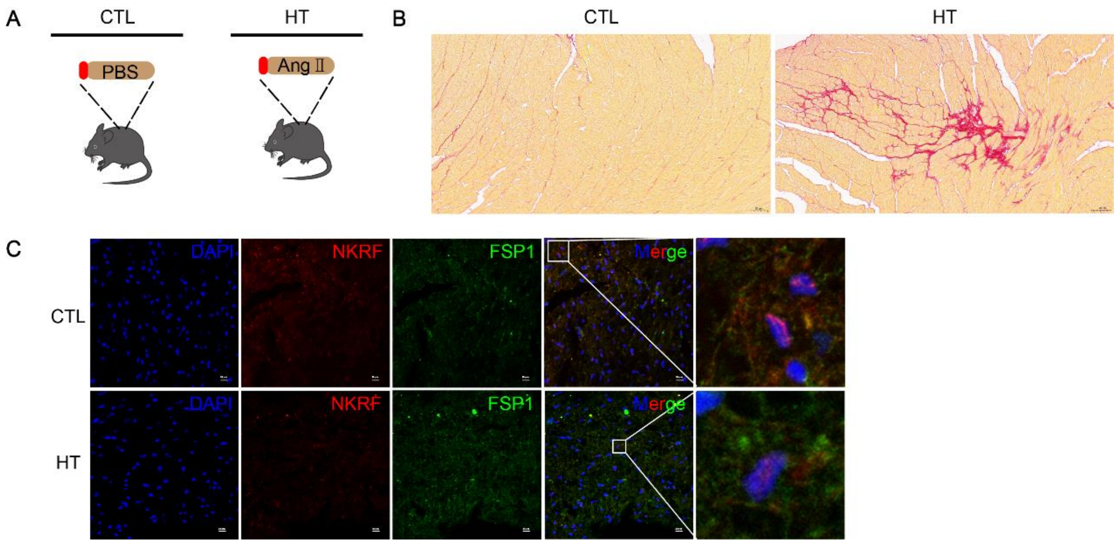

Figure S2. NKRF was downregulated in CFs in a hypertensive mice model.

A, Schematic diagram of the Ang II-induced hypertensive C57BL/6J mouse model. **B**, Picrosirius red staining shows that the ECM of the myocardium has a significant increase in collagen in hypertensive mice treated with Ang II (400 ng kg^{-1} min^{-1}) for 4 weeks (scale bar = 50 µm). **C**, Immunofluorescence colocalization staining of NKRF (red) and FSP1 (green) in transverse heart cross sections obtained from C57BL/6J mice treated with Ang II (scale bar = 20 µm). ECM, extracellular matrix; CFs, cardiac fibroblasts; Ang II, angiotensin II; CTL, control; HT, hypertension; FSP1, fibroblast-specific protein 1.

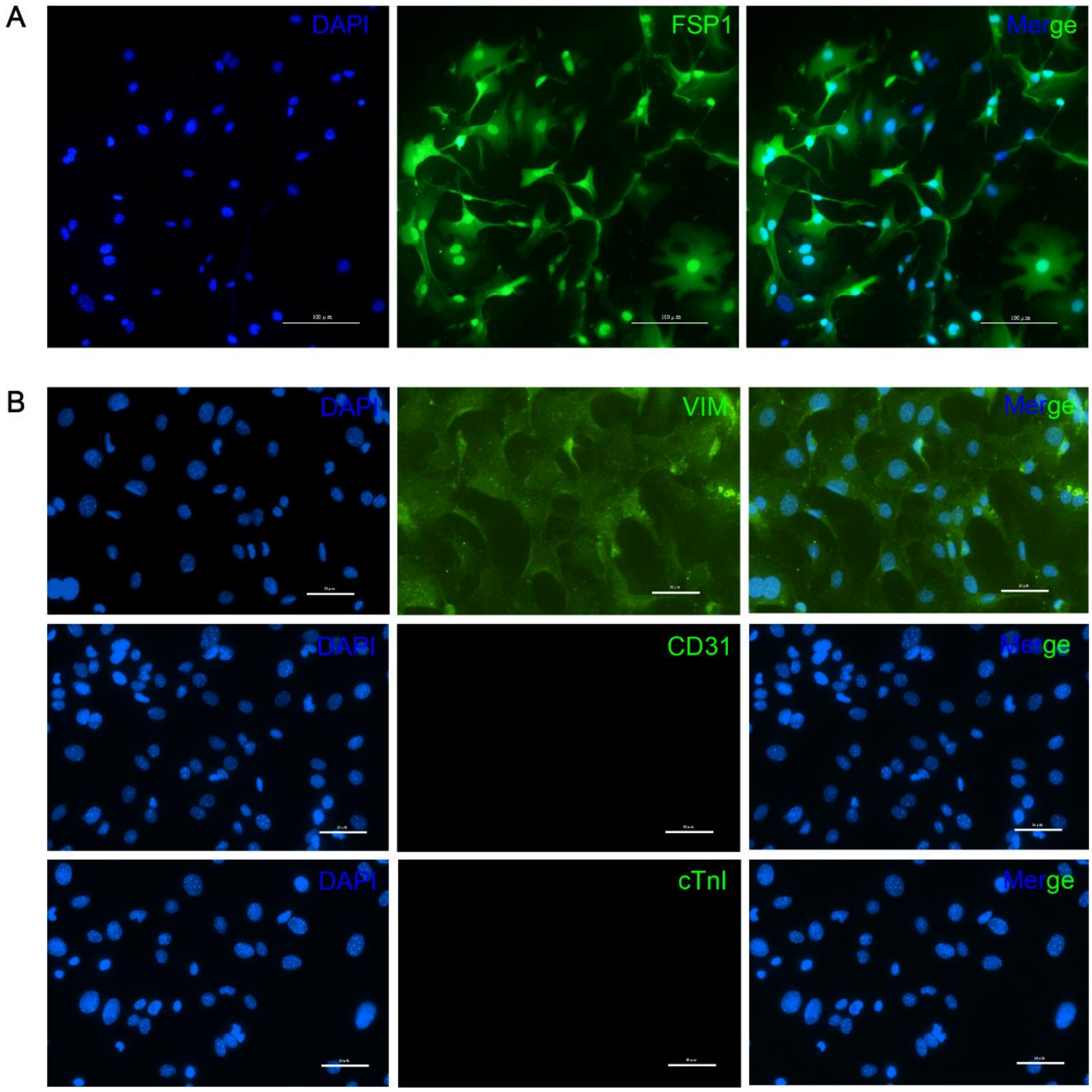

Figure S3. Immunofluorescence staining identifies the purity of the isolated primary CFs from neonatal C57BL/6J mice (1–3 days old).

A, The isolated primary CFs expressed the CF-specific marker protein FSP1 (green) shown using a Zeiss confocal laser scanning microscope (scale bar = 100 µm). **B**, The isolated primary CFs expressed another CF-specific marker protein, VIM, but not the endothelial cell marker protein CD31 and cardiomyocyte marker protein cTnI (scale bar = 50 µm). CFs, cardiac fibroblasts; FSP1, fibroblast-specific protein 1; VIM, vimentin; cTnI, cardiac troponin I.

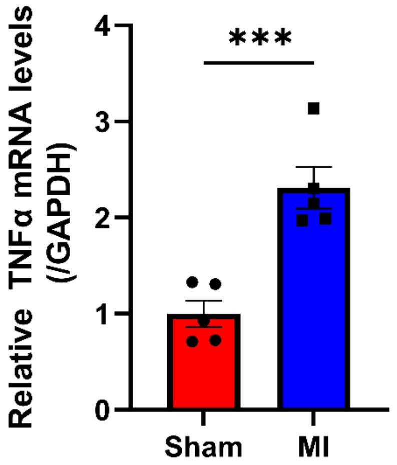

Figure S4. Gene expression levels of TNF-α in the hearts of mice after myocardial infarction (MI) and sham operation.

TNF-α mRNA levels were significantly elevated in the infarct border zone of the MI group (n = 5) compared to the sham group (n = 5) at 3 days post-MI. Data are presented as the mean ± SEM. ***P < 0.001 by unpaired two-tailed Student's t-test.

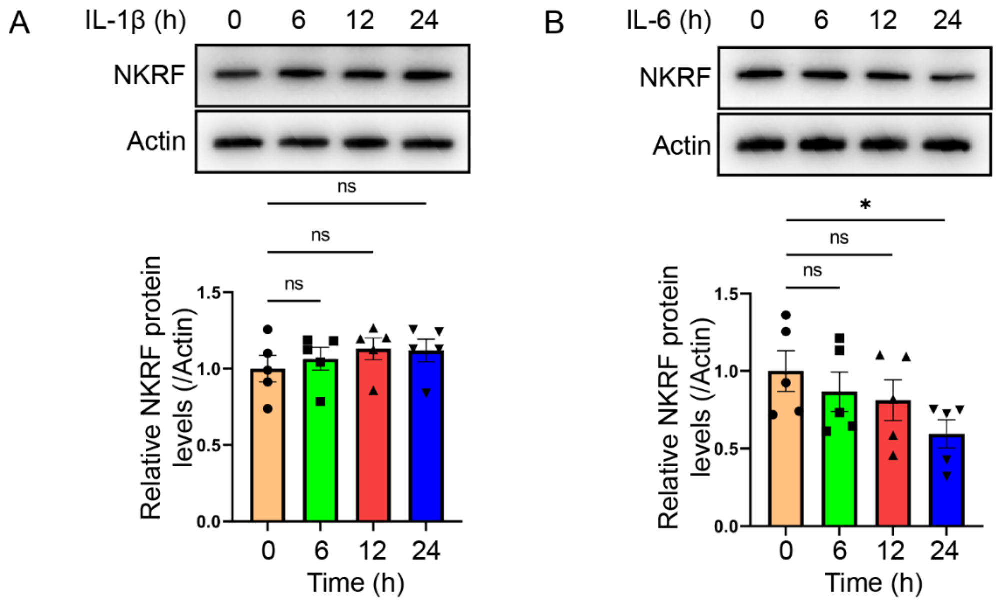

Figure S5. Effects of IL-1β and IL-6 on NKRF expression in cardiac fibroblasts.
Western blot analysis showing no significant effect on NKRF expression with IL-1β (10 ng mL^{-1}) treatment (**A**, n=5), while IL-6 (20 ng mL^{-1}) induced a time-dependent downregulation of NKRF expression (**B**, n=5) in cardiac fibroblasts. Data are the

mean ± SEM. P-values were determined using one-way ANOVA with Bonferroni multiple comparisons test (A and B). NS, non-significant and *P<0.05.

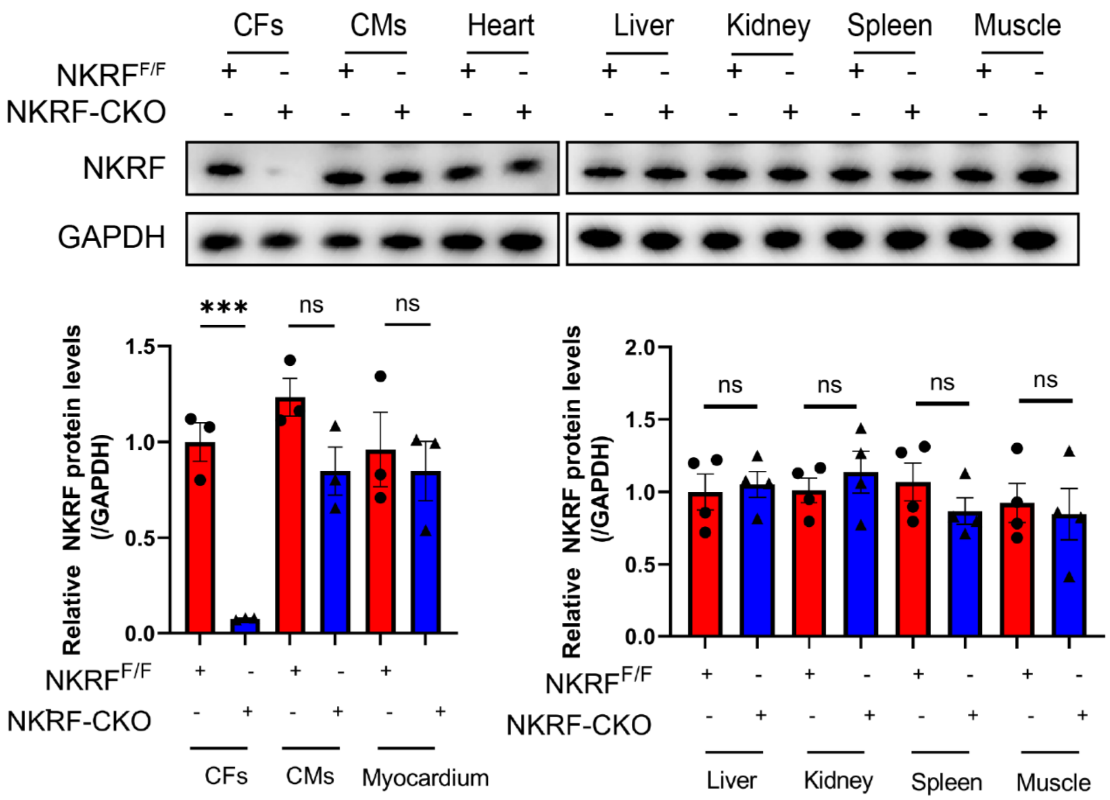

Figure S6. Western blotting identified the expression of NKRF in multiple cells and tissue in NKRF$^{F/F}$ and NKRF-CKO mice.

The proteins from CFs, CMs, total heart homogenates, liver, kidney, spleen, and muscle homogenates were subjected to western blotting (**upper panel**) and quantified (**lower panel**) (n = 3). NKRF$^{F/F}$, NKRF$^{flox/flox}$ mice; NKRF-CKO, NKRF$^{flox/flox}$:Cre^{S100a4} mice; CFs, cardiac fibroblasts; CMs, cardiac myocytes.

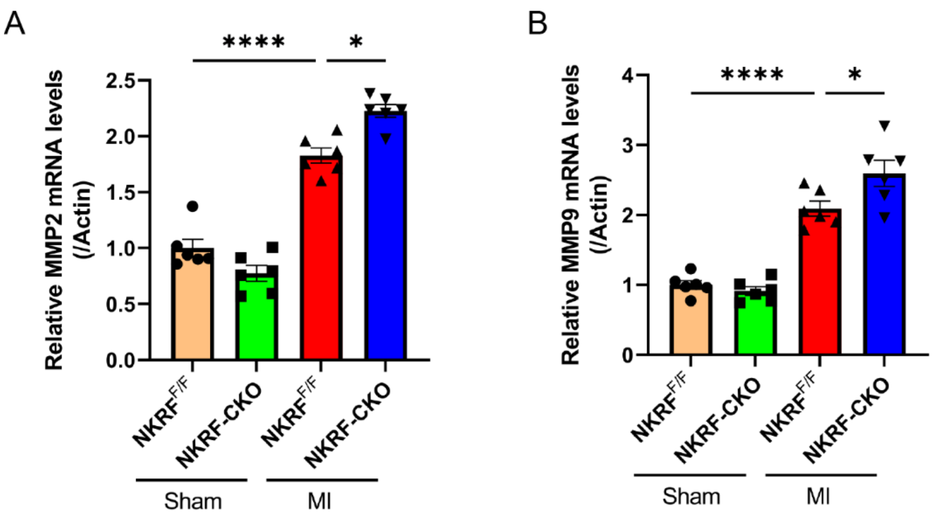

Figure S7. Expression analysis of *Mmp2* and *Mmp9* mRNAs in the border region of myocardial infarction (MI).

Quantification of *Mmp*2 (**A**, n = 6) and *Mmp*9 (**B**, n = 6) mRNA expression in the infarct border region of NKRF$^{F/F}$ and NKRF-CKO mice. Data are expressed as the mean ± SEM. *P<0.05 and ****P<0.0001 by two-way ANOVA with Bonferroni multiple comparisons test. NKRF$^{F/F}$ and NKRF-CKO, NKRF$^{flox/flox}$ and NKRF$^{flox/flox}$:Cre^{S100a4} mice.

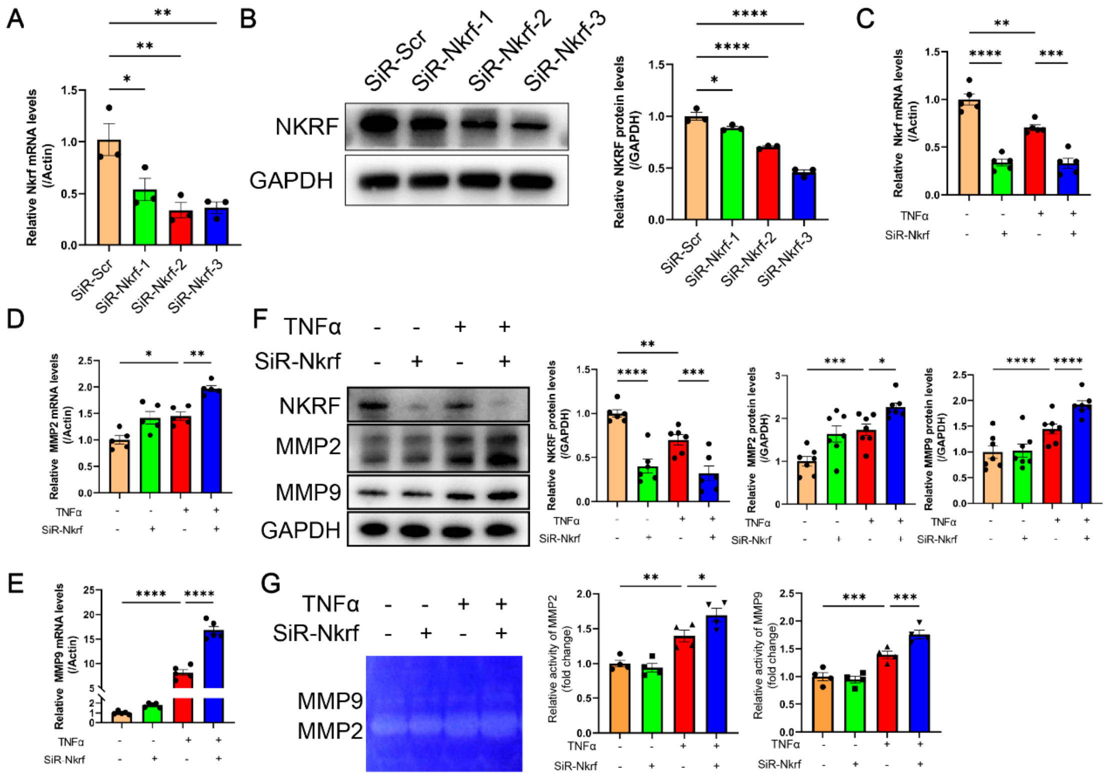

Figure S8. NKRF knockdown enhances TNF-α-induced expression of MMP2 and MMP9 in CFs.

A and B, Verification of knockdown efficiency of three SiR-*Nkrf* at mRNA (A) and protein (B) levels (n = 3). **C–G**, Primary CFs were transfected with SiR-*Nkrf* or SiR-Scr for 24 h, followed by treatment with TNF-α (10 ng mL^{-1}) for another 24 h. **C–E**, Relative expression of *Nkrf* (C), *Mmp2* (D), and *Mmp9* (E) at the mRNA level. The values were first normalized to actin and then calculated as fold changes vs. CFs transfected with SiR-Scr and treated with PBS. **F**, Relative expression of NKRF, MMP2, and MMP9 at the protein level. The values were first normalized to GAPDH, then calculated as fold changes vs. CFs transfected with SiR-Scr and treated with PBS. **G**, Representative images of gelatin zymography and quantitative analysis of MMP2 and MMP9 activities (n = 4). Samples were obtained from the supernatant of the treated CFs. Data are the mean ± SEM. P-values were determined using unpaired two-tailed Student's t-test (A and B) and two-way ANOVA with Bonferroni multiple comparisons test (C, D, E, F, and G). *P<0.05, **P<0.01, ***P<0.001, and ****P<0.0001. CFs, cardiac fibroblasts; SiR-*Nkrf*, Nkrf siRNA (small interfering RNA); SiR-Scr, scrambled siRNA.

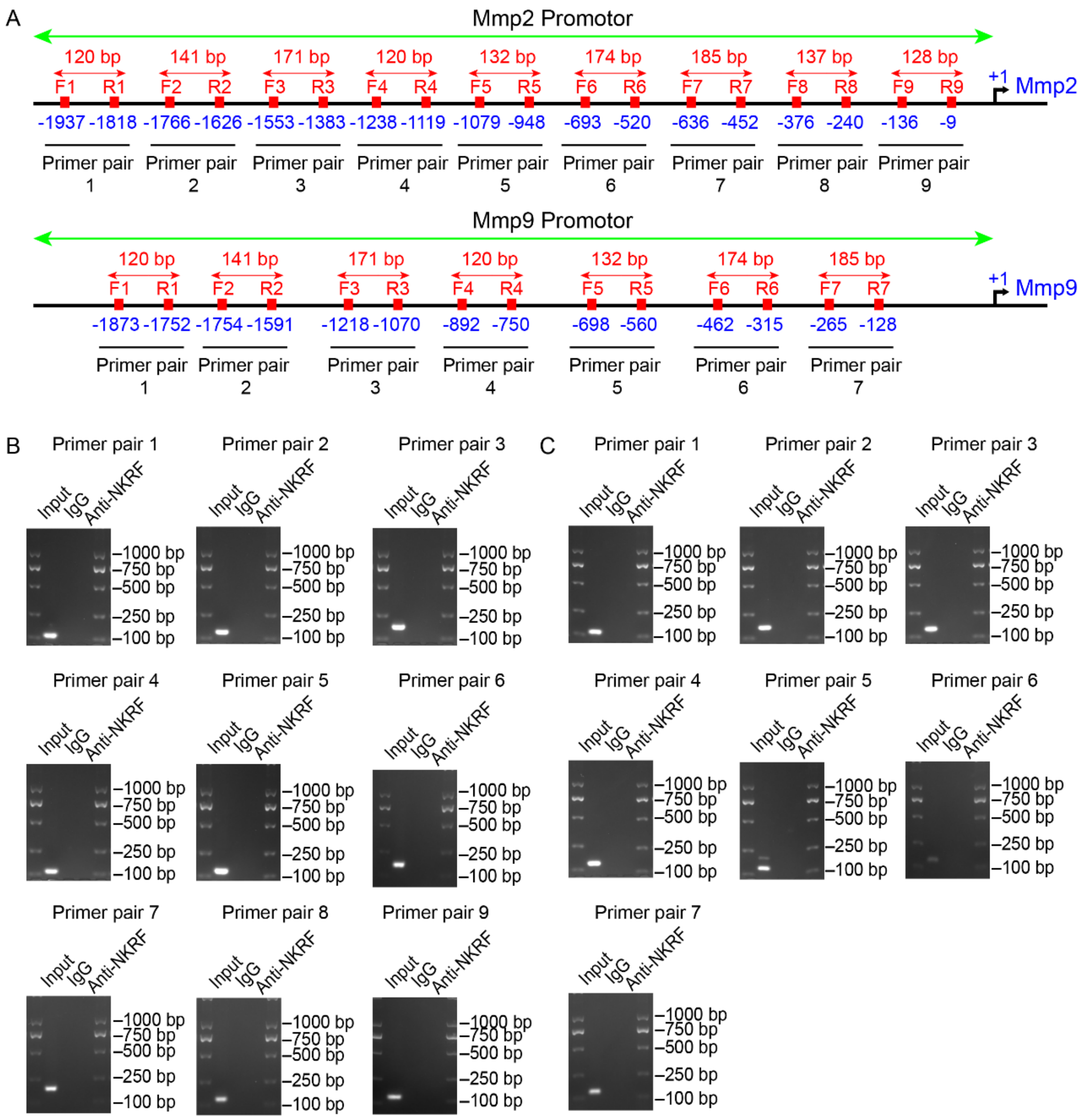

Figure S9. ChIP experiments using NKRF antibody enriched *Mmp2* and *Mmp9* gene promoter regions as templates in CFs.

A, Schematic diagram of primer pairs covering the gene promoter regions of *Mmp2* and *Mmp9*. **B and C**, ChIP agarose gel electrophoresis results using NKRF antibody enriched different *Mmp2* (**B**) and *Mmp9* (**C**) gene promoter regions as templates in CFs. CFs, cardiac fibroblasts; ChIP, chromatin immunoprecipitation.

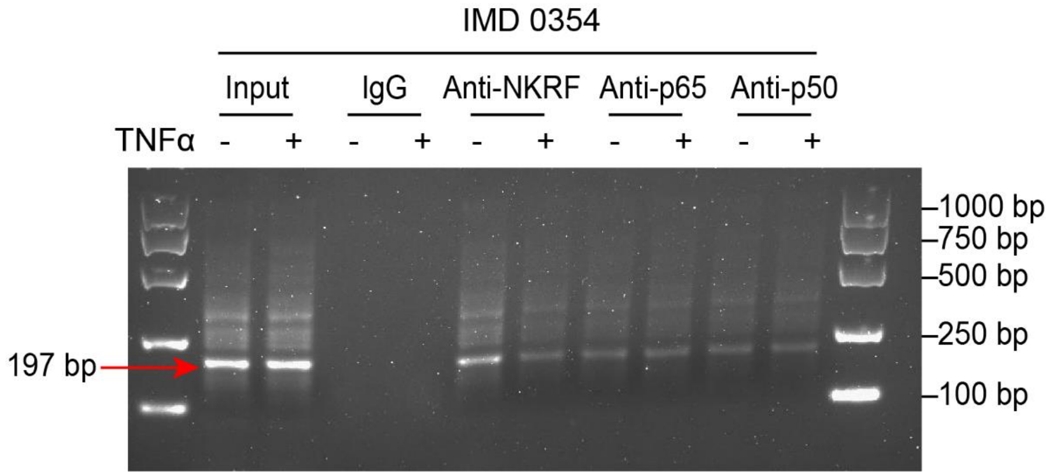

Figure S10. Agarose gel electrophoresis results using the NRE in HuR promotor region as a template enriched by anti-NKRF, anti-p65, and anti-p50 antibody in ChIP experiments.

Primary cardiac fibroblasts were pretreated with the inhibitor of the NF-κB pathway (5 μM IMD 0354) for 1 h, then treated with TNF-α (10 ng mL^{-1}) or PBS for another 24 h. TNF-α-induced enrichment of p65 and p50 to the NRE region within the *HuR* promoter was inhibited after blocking the NF-κB pathway by IMD 0354, but this did not affect the downregulation trend of NKRF enrichment in the NRE region within the *HuR* promoter. ChIP, chromatin immunoprecipitation; NRE, negative regulatory element.

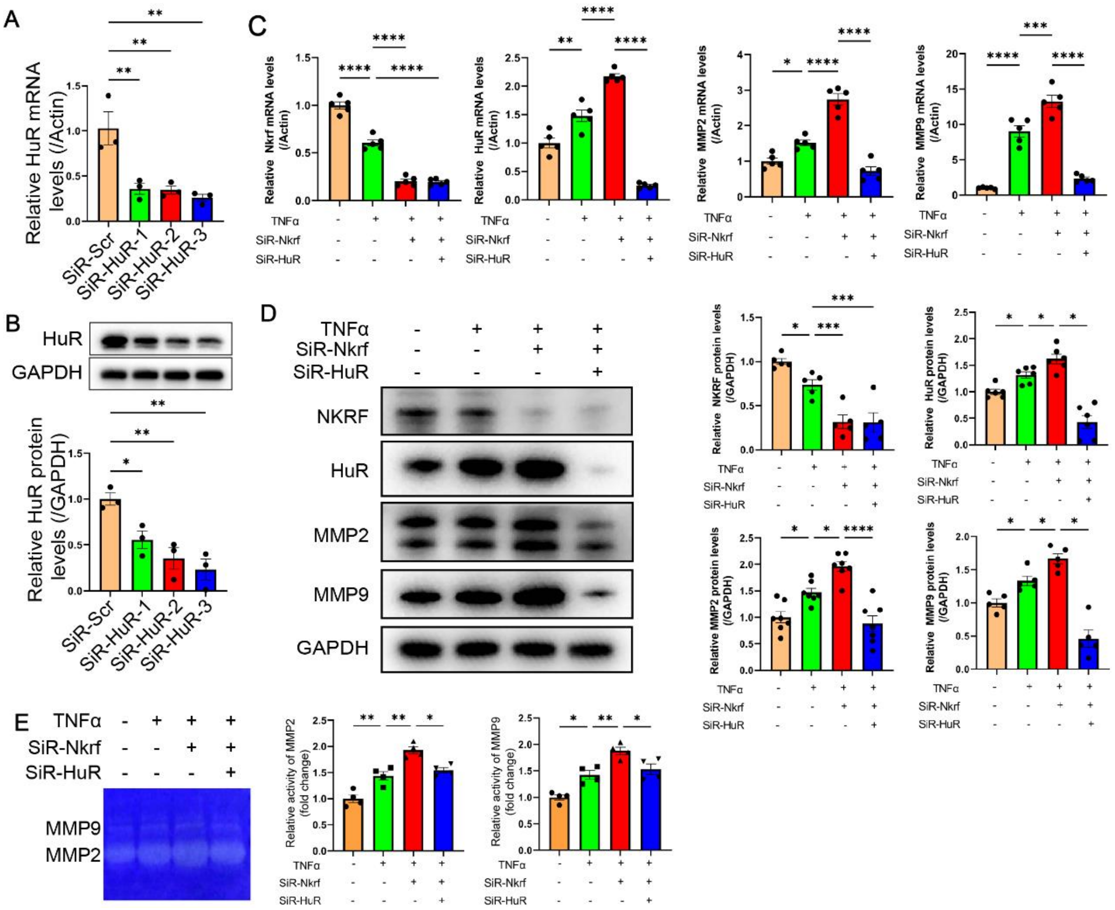

Figure S11. HuR knockdown reverses the promoting effect of NKRF knockdown on the expression of MMP2 and MMP9 in CFs.

A and B, Verification of knockdown efficiency of three SiR-*HuR* at mRNA (A) and protein (B) levels (n = 3). **C–E**, Primary CFs were transfected with SiR-*Nkrf* and SiR-*HuR* for 24 h followed by treatment with TNF-α (10 ng mL^{-1}) for another 24 h. **C and D**, Relative expression of *Nkrf*, *HuR*, *Mmp2,* and *Mmp9* at the mRNA (C) and protein (D) levels. The values were first normalized to actin (at the mRNA level) or GAPDH (at the protein level) and then calculated as fold changes vs. CFs transfected with SiR-Scr and treated with PBS. **E,** Representative images of gelatin zymography and quantitative analysis of MMP2 and MMP9 activities (n = 4). Samples were obtained from the supernatant of the treated CFs. Data are the mean ± SEM. P-values were determined using unpaired two-tailed Student's t-test (A and B) and one-way ANOVA with Bonferroni multiple comparisons test (C, D, and E). *P<0.05, **P<0.01, ***P<0.001, and ****P<0.0001. CFs, cardiac fibroblasts; SiR-*Nkrf*, *Nkrf* small interfering RNA (siRNA); SiR-*HuR*, *HuR* siRNA; SiR-Scr, scrambled siRNA.

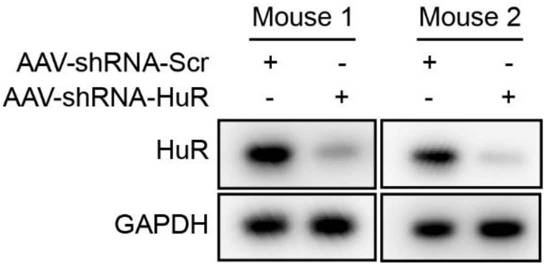

Figure S12. Western blotting verifies the knockdown efficiency of HuR in CFs isolated from two NKRF-CKO mice treated with AAV-shRNA-*HuR*.

HuR expression significantly decreased in CFs isolated from two NKRF-CKO mice treated with AAV-shRNA-*HuR* for 14 days. CFs, cardiac fibroblasts; NKRF-CKO mice, $NKRF^{flox/flox}$:Cre^{S100a4} mice; AAV-shRNA-*HuR*, adeno-associated virus short hairpin RNA-HuR; AAV-shRNA-Scr, adeno-associated virus short hairpin RNA-scramble control.

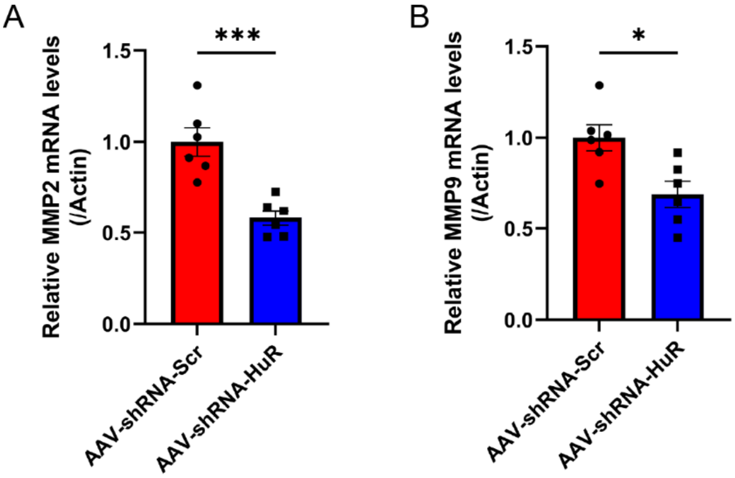

Figure S13. HuR knockdown inhibits the expression of MMP2 and MMP9 mRNAs post-MI in NKRF-CKO mice.

Quantitative analysis of MMP2 (**A**, n=6) and MMP9 (**B**, n=6) mRNAs in the border region of MI in NKRF-CKO mice receiving AAV-shRNA-*HuR* or AAV-shRNA-Scr treatment. Data are the mean ± SEM. *P<0.05 and ***P<0.001 by unpaired two-tailed Student's t-test. NKRF-CKO mice, NKRF$^{flox/flox}$:Cre^{S100a4} mice; MI, myocardial infarction; AAV-shRNA-*HuR*, adeno-associated virus short hairpin RNA-*HuR*; AAV-shRNA-Scr, adeno-associated virus short hairpin RNA-scramble control.

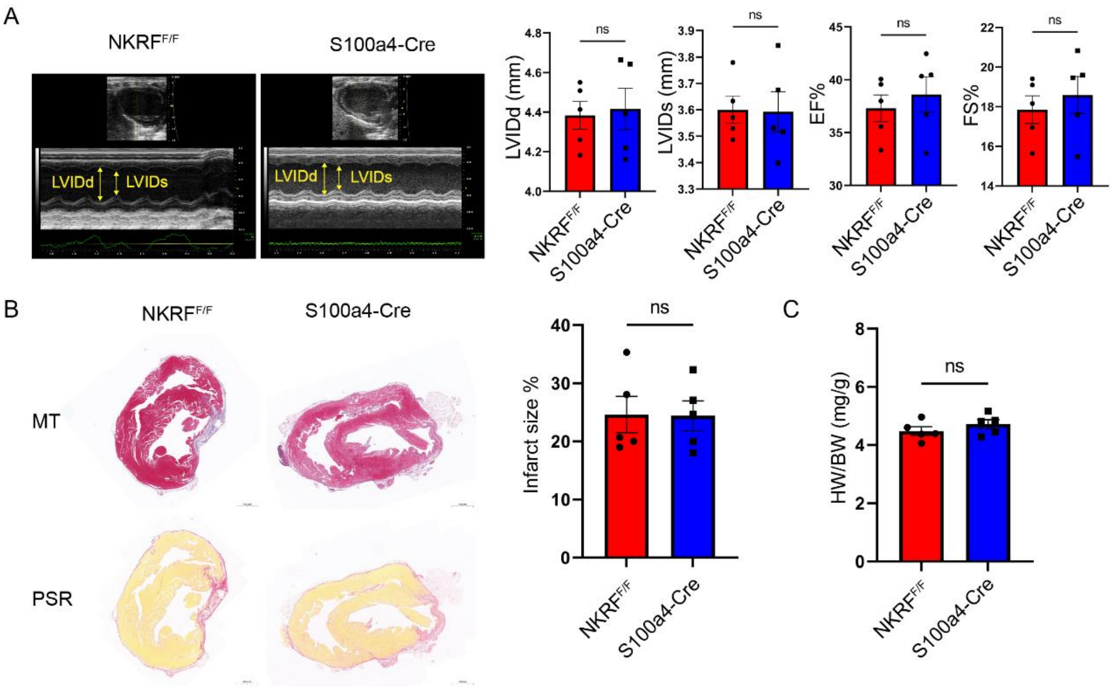

Figure S14. Assessment of cardiac function, infarct size, and heart weight to body weight (HW/BW) ratio in S100a4-Cre and NKRF$^{F/F}$ mice at 4 weeks post-myocardial infarction.

A. Echocardiography and measured LVIDd, LVIDs, EF%, and FS% in S100a4-Cre and NKRF$^{F/F}$ mice (n = 5). **B**. Masson's Trichrome (MT) and Picrosirius Red (PSR) staining from

heart transverse cross-sections obtained from S100a4-Cre and NKRF$^{F/F}$ mice, along with quantification of infarct size (scale bar = 1000 μm, n = 5). **C**. The HW/BW ratio of S100a4-Cre and NKRF$^{F/F}$ mice (n = 5). Data are expressed as the mean ± SEM. NS, non-significant by unpaired two-tailed Student's t-test. EF, left ventricular ejection fraction; FS, fractional shortening; LVIDd, left ventricular internal diastolic dimension; LVIDs, left ventricular internal systolic dimension; NKRF$^{F/F}$, NKRF$^{flox/flox}$ mice.

Supplemental tables

Table S1. Demographic and clinical information of STEMI patients and healthy donors recruited for cytokine measurement.

Characteristic	Overall (n=23)	Control (n=12)	STEMI (n=11)	P-value [a)]
Age (year)	62.91 ± 2.97	62.25 ± 4.11	63.64 ± 4.49	0.82
Male sex, n (%)	15（65.22）	7（58.33）	8（72.72）	0.67
Body mass index (kg m^{-2})	25.69 ± 0.48	24.98 ± 0.61	26.46 ± 0.70	0.12
Systolic blood pressure (mm Hg)	125.30 ± 4.18	125.20 ± 4.80	125.40 ± 7.25	0.98
Diastolic blood pressure (mm Hg)	74.61 ± 3.27	75.83 ± 4.38	73.27 ± 5.08	0.71
Hypertension, n (%)	10 (43.48)	3 (25)	7 (63.64)	0.10
Type 2 diabetes, n (%)	9（39.13)	4 (33.33)	5 (45.45)	0.68
Dyslipidemia, n (%)	6 (26.09)	1 (8.33)	5 (45.45)	0.07
Smoking (active or former), n (%)	7 (30.43)	3 (25)	4 (36.36)	0.67
History of stroke, n (%)	0 (0)	0 (0)	0 (0)	>0.99
History of CABG, n (%)	0 (0)	0 (0)	0 (0)	>0.99
History of carcinoma, n (%)	2 (8.70)	1 (8.33)	1 (9.09)	>0.99
Laboratory parameters				
NT-proBNP (pg mL^{-1}), median (IQR)	232 (39.38–586.70)	66.22 (35.75–165.0)	586.70 (351.50–2330)	<0.01
Creatine kinase (U L^{-1}), median (IQR)	88 (58–202)	62.50 (52–104.80)	202 (64–546)	0.03
Cardiac troponin I (ng L^{-1}), median (IQR)	19.05 (3.44–4053)	4.51 (2.60–7.12)	4053 (2668–14415)	<0.01
Total cholesterol (mmol L^{-1}), median (IQR)	4.34 (3.25–4.77)	3.68 (3.05–4.44)	4.64 (4.30–4.93)	0.04

LDL-cholesterol, (mmol L^{-1}), median (IQR)	2.51 (1.74–3.11)	2 (1.58–2.50)	3.04 (2.69–3.59)	0.02
HDL-cholesterol (mmol L^{-1})	1.08 ± 0.08	1.24 ± 0.08	0.91 ± 0.11	0.03
Alanine aminotransferase (U L^{-1})	37.22 ± 6.44	22.67 ± 4.15	53.09 ± 11.05	0.01
Aspartate aminotransferase (U L^{-1})	33.17 ± 5.03	20.08 ± 1.68	47.45 ± 8.59	<0.01
Gamma glutamyltransferase (U L^{-1})	37.30 ± 4.89	37.42 ± 6.60	37.18 ± 7.58	0.98
Urea nitrogen (mmol L^{-1})	5.42 ± 0.20	5.43 ± 0.31	5.41 ± 0.24	0.97
Creatinine (μmol L^{-1})	71 ± 2.66	70.50 ± 3.95	71.55 ± 3.69	0.85

STEMI, ST-segment elevation myocardial infarction. Data are expressed as the mean ± standard error of the mean (SEM) or median (interquartile range [IQR]) [a)], P values were calculated using unpaired two-tailed Student’s t-test for the comparison between STEMI and control groups.

Table S2. Primer sequences used in RT-PCR.

Gene	Primer Sequence (5′-3′)
Nkrf<br>(Mus musculus)	Forward:<br>CCACGGAGCTGACCAATGAT<br>Reverse:<br>CCGTTTTCGACCTACCACCA
HuR<br>(Mus musculus)	Forward:<br>TGGGCGAATCATCAACTCCA<br>Reverse:<br>CGGATAAAGGCAACCCCTCT
Mmp2<br>(Mus musculus)	Forward:<br>GATAACCTGGATGCCGTCGTG<br>Reverse:<br>CTTCACGCTCTTGAGACTTTGGTTC
Mmp9<br>(Mus musculus)	Forward:<br>TAGATCATTCCAGCGTGCCG<br>Reverse:<br>GCTTAGAGCCACGACCATACA
TNF-α	Forward:

(Mus musculus)	AGCCGATGGGTTGTACCTTG<br>Reverse:<br>ATAGCAAATCGGCTGACGGT
Firefly luciferase	Forward:<br>TCAAAGAGGCGAACTGTGTG<br>Reverse: GTGTTCGTCTTCGTCCCAGT
Renilla luciferase	Forward:<br>ATAACTGGTCCGCAGTGGTG<br>Reverse:<br>TAAGAAGAGGCCGCGTTACC
HuR Promotor<br>(Mus musculus)	Forward:<br>AGCCTATGCTGGCCTTGAAC<br>Reverse: GCAAGCCACTTCTCCTTCCT
Actin<br>(Mus musculus)	Forward:<br>GGCTGTATTCCCCTCCATCG<br>Reverse:<br>CCAGTTGGTAACAATGCCATGT
Mmp2 Promotor (Primer pair 1)<br>(Mus musculus)	Forward:<br>CTCTCTGTTCCCTGTGTGCTT<br>Reverse:<br>ACTCTCGTCCCTGGTAAGTGT
Mmp2 Promotor (Primer pair 2)<br>(Mus musculus)	Forward:<br>ACTGTTCCTAAAGGCGCTAGAAT<br>Reverse:<br>AATTCCAGGAACCCCAAACCTT
Mmp2 Promotor (Primer pair 3)<br>(Mus musculus)	Forward:<br>CAGATCACATTCCTCTCGCCA<br>Reverse:<br>TACCACCTGGTCAGGGATTCA
Mmp2 Promotor (Primer pair 4)<br>(Mus musculus)	Forward:<br>TCAAAATGCTATTCAGCCCACA<br>Reverse:<br>CACCCAGTCTTGTGGATAGTCA

Mmp2 Promotor (Primer pair 5) (Mus musculus)	Forward: CCTCCCCAGAGGTCCTTTCTA Reverse: GCTGTAGAGTGGGTTAGCCAG
Mmp2 Promotor (Primer pair 6) (Mus musculus)	Forward: ACTCTGTTCAGGCAGGTGATG Reverse: GCAGAACACACATTTCCAGCA
Mmp2 Promotor (Primer pair 7) (Mus musculus)	Forward: ACTCAGAAGTCACATCGTCCAAG Reverse: CTTGGTTTCACTGGTGGTCCTC
Mmp2 Promotor (Primer pair 8) (Mus musculus)	Forward: TTCCTTGAGTGGCTCTATGGC Reverse: GGGATACTGGAATCAGGACGC
Mmp2 Promotor (Primer pair 9) (Mus musculus)	Forward: CCAGTTAGGGAGCAAGAAGGG Reverse: GTGGGGAAGTGAGGTCCGA
Mmp9 Promotor (Primer pair 1) (Mus musculus)	Forward: AAATGGCCAAGTGGGTCAGAG Reverse: CCATCTGTCAGTGTTGGAGGT
Mmp9 Promotor (Primer pair 2) (Mus musculus)	Forward: TGGAGACTCTGAGACAGGGTT Reverse: ACACACATCCATGACTGCCAA
Mmp9 Promotor (Primer pair 3) (Mus musculus)	Forward: TGGTTGGGAAATGACGAGGTT Reverse: CATGTGCTTCCCAACGAACAG
Mmp9 Promotor (Primer pair 4) (Mus musculus)	Forward: ATGACGATCTCACAGCTCGG

	Reverse: CCACTTTCAGCAGTGTTCACG
Mmp9 Promotor (Primer pair 5) (Mus musculus)	Forward: AAGGCTTGAGCGACAAAGGG<br>Reverse: TGGGGAAGGAAAGATGAGCC
Mmp9 Promotor (Primer pair 6) (Mus musculus)	Forward: CAAAGACTCTATCAGGGGGCG<br>Reverse: GCCCAGTAAAAGACTGGGAGA
Mmp9 Promotor (Primer pair 7) (Mus musculus)	Forward: CACACCCTCCTTCCCTTTCC<br>Reverse: ACTACCCCCTCCCTTATGCC
